# Enhanced clinical outcomes with radiotherapy in diagnostically challenging intracranial plasmacytomas: Analysis of 190 cases

**DOI:** 10.1002/cam4.7017

**Published:** 2024-03-08

**Authors:** Yuan Feng, Zongpu Zhang, Fufang Qiu, Zixiao Yang, Ji Xiong, Wei Zhu, Fangzhu Wan, Bobin Chen, Jiguang Wang, Yi Zhang, Wei Hua

**Affiliations:** ^1^ Department of Neurosurgery, Huashan Hospital Fudan University Shanghai China; ^2^ National Center for Neurological Disorders Shanghai China; ^3^ Shanghai Key Laboratory of Brain Function Restoration and Neural Regeneration Shanghai China; ^4^ Neurosurgical Institute of Fudan University Shanghai China; ^5^ Shanghai Clinical Medical Center of Neurosurgery Shanghai China; ^6^ Department of Pathology, Huashan Hospital Fudan University Shanghai China; ^7^ Department of Radiation Oncology, Shanghai Proton and Heavy Ion Center Fudan University Cancer Hospital Shanghai China; ^8^ Shanghai Key Laboratory of Radiation Oncology (20dz2261000) Shanghai China; ^9^ Department of Hematology, Huashan Hospital Fudan University Shanghai China; ^10^ Division of Life Science, Department of Chemical and Biological Engineering, and State Key Laboratory of Molecular Neuroscience The Hong Kong University of Science and Technology Hong Kong SAR China; ^11^ SIAT‐HKUST Joint Laboratory of Cell Evolution and Digital Health HKUST Shenzhen‐Hong Kong Collaborative Innovation Research Institute Shenzhen China; ^12^ Hong Kong Center for Neurodegenerative Diseases, InnoHK Hong Kong SAR China

**Keywords:** intracranial plasmacytomas, multiple myeloma, prognostic factors, progression, radiotherapy

## Abstract

**Background:**

Intracranial plasmacytomas are rare tumors arising from plasma cells with approximately half of the cases progressing to multiple myeloma (MM). However, there is a lack of comprehensive clinical cohort analysis on the clinical and pathological features, progression, and outcomes of intracranial plasmacytomas.

**Methods:**

A retrospective analysis of 190 cases was conducted, combining data from 38 cases in a single institution and 152 cases from the literature. Patient demographics, clinical presentations, tumor locations, imaging features, surgical treatments, and follow‐up outcomes were collected and analyzed. Survival analysis and Cox regression analysis were performed to identify prognostic factors.

**Results:**

A total of 190 intracranial plasmacytoma patients with an average age of 55.4 years were included in the study. The preoperative misdiagnosis ratio was high at 55.3%, and 59.7% of the tumors affected the calvaria convexity, compared to 40.3% located at the skull base. Resection and biopsy were achieved in 72.4% and 27.6% patients, respectively. Among them, 34.2% (65/190) of patients were initially diagnosed with MM with intracranial plasmacytoma as their first presentation (MM‐IPFP), while 63.2% (120/190) of patients were diagnosed with solitary intracranial plasmacytoma (SIP), including 61 extramedullary plasmacytomas and 59 solitary bone plasmacytomas. In the SIP group, 22.4% (24/107) of patients experienced disease progression leading to the development of MM during a median follow‐up time of 42.6 months (range 1–230 months). Multivariate analysis unveiled that radiotherapy (HR, 0.05; 95% CI, 0.00–0.87; *p* = 0.04), not surgery, was a protective prognostic factor for overall survival in MM‐IPFP patients. Comparison between the SIP progression group and non‐progression group revealed a significant difference of Ki‐67 index (non‐progression vs. SIP progression, 8.82% ± 7.03 vs. 16.5% ± 10.5, *p* < 0.05). AUC analysis determined that a cutoff value of 9.0% was the best predictor of SIP progression, with an area under the curve of 0.712.

**Conclusions:**

This retrospective clinical analysis highlights the potential role of radiotherapy, rather than surgical resection, in improving the outcomes of intracranial plasmacytoma. Additionally, the Ki‐67 index is identified as a valuable marker for predicting disease progression. This would provide some evidence for the paradigm of diagnosis and treatment modalities for intracranial plasmacytomas from the large cohort.

## 1 INTRODUCTION

Intracranial plasmacytomas are uncommon tumors characterized by an abnormal proliferation of clonal plasma cells that secrete immunoglobulins. These neoplasms can manifest as solitary bone plasmacytoma (SBP), extramedullary plasmacytoma (EMP), or multiple lesions.^1^, ^2^ Nearly 5% of patients with multiple myeloma (MM) present an initial diagnosis of solitary plasmacytoma, with EMP commonly occurring in the head and neck and SBP affecting in the axial skeleton without systemic involvement.^3^ Solitary intracranial plasmacytoma (SIP) generally has a favorable prognosis, with a 5‐year overall survival rate of 76.1%.^4^ However, the high misdiagnosis rate and susceptibility of progression to MM pose significant challenges in the management and treatment of this rare disease. Intracranial plasmacytoma commonly involves the skull base, meninges, and brain but lacks specific imaging features. Approximately 25% of solitary plasmacytoma cases progress to MM within 5 years,^4^ with a median time to progression of 10.5 months for SBP and 18.6 months for EMP.^5^ Systemic examinations are necessary to monitor disease progression, as prognosis significantly worsens when accompanied by MM, resulting in a median survival time of merely 6.7 months.^4^ Early identification of patients with plasmacytomas and those at risk of progressing to MM is crucial for appropriate management.

The treatment of intracranial plasmacytoma requires multidisciplinary guidance, particularly when a diagnosed of MM is combined with plasmacytoma. The benefit of surgery in improving outcomes remains controversial and is recommended primarily for tumors causing structural instability or neurological compromise.^4^, ^6^ For solitary plasmacytomas, radiotherapy (RT) is the preferred intervention, reducing local relapse rates from 60% to 12%.^6^, ^7^ However, the optimal radiation dose for treatment remains uncertain and should be judiciously adjusted for patients with progression to MM. The current standard chemotherapy (CMT) regimen for newly diagnosed MM involves dexamethasone, proteasome inhibitors (bortezomib or carfilzomib), and lenalidomide. The combination improves the rate of complete response to 30% and prepares patients for autologous hematopoietic cell transplant.^8^ Adjuvant CMT has also demonstrated benefits in preventing the evolution of SBP to MM.^9^ Meanwhile, novel immunotherapy options such as chimeric antigen receptor T‐cell therapy^10^ and bispecific antibodies^11^ offer promising approaches for clinical trials. Understanding molecular events including cytogenetic abnormalities correlated with treatment response,^12^ will further facilitate the implementation of individualized modalities to optimize patient outcomes.

In this retrospective study, we analyzed the characteristics and outcomes of 190 patients with intracranial plasmacytoma, combining data from 38 patients at our center and 152 patients from a literature review. The aim is to identify clinical characteristics and prognostic factors associated with patient outcomes and determine the best treatment and post‐operative regime for this population.

## 2 METHODS

### 2.1 Retrospective clinical cohort design and data collection

Medical data from patients admitted to the Department of Neurosurgery, Huashan Hospital, between January 1st, 2002, and October 31st, 2022, was screened. The inclusion criteria were as follows: (1) patients aged >18 and < 80 years old; (2) histopathology confirmation of intracranial plasmacytomas; (3) availability of complete clinical and pathological data; and (4) availability of survival and follow‐up data. The exclusion criteria included: (1) lack of available clinical data; (2) absence of histopathological confirmation; (3) difficulty in patients or family to cooperation with follow‐up; and (4) preexisting diagnosis of MM prior to surgery. Patients diagnosed with MM during their post‐operation hospitalization period by bone marrow biopsy were still included in this study and classified as patients with MM with intracranial plasmacytoma as the first presentation (MM‐IPFP). A total of 38 patients were enrolled in this study. This cohort research was approved by the Institutional Review Board of the hospital (approval ID: 2015–256) and was conducted in accordance with the Helsinki Declaration. Written informed consent was obtained from all patients. This work was reported in line with the Strengthening the Reporting of Cohort Studies in Surgery (STROCSS) criteria and was retrospectively registered at https://www.chictr.org.cn. Electronic medical records were used to collect clinical data, including basic information, clinical symptoms, imaging findings, surgical findings and outcomes, pathological results, and clinical outcomes.

### 2.2 Histopathological diagnosis and immunohistochemistry

The tumor tissue specimens were fixed in a 10% formalin solution, followed by embedding in paraffin and staining with hematoxylin–eosin. The diagnosis of solitary plasmacytoma and MM was determined based on the established International Myeloma Working Group (IMWG) criteria.^2^ For immunohistochemistry, a representative tissue block was selected and subjected to the avidin‐biotin complex horseradish‐peroxidase method. Primary antibodies against CD138 (Abcam, ab130405), kappa light chain (Abcam, ab227654), lambda light chain (Abcam, ab195573), and Ki‐67 (Abcam, ab15580) were applied according to the manufacturer's instructions. Two pathologists independently evaluated all stained sections without any knowledge of the corresponding clinical data. In cases of discordance, the staining results were reviewed by the observers until a consensus was reached.

### 2.3 Clinical management of intracranial plasmacytoma

Patients diagnosed with intracranial plasmacytoma were managed according to a multidisciplinary approach. The treatment strategy involved a combination of surgical intervention, RT, and CMT. Surgical resection was considered for accessible and resectable lesions, aiming to achieve maximal tumor removal while preserving neurological function. The extent of tumor resection was classified as total resection (no residual tumor), subtotal resection (more than 90% of tumor removed), partial resection (less than 90% of tumor removed), and biopsy based on the neurosurgeon's intraoperative estimate. RT was employed as a primary treatment modality or as an adjuvant therapy post‐surgery. Briefly, a total of fractionated 30–60 Gy irradiation was conducted for solitary plasmacytoma and low‐dose RT (8 Gy × 1 fraction or 10–30 Gy in 2.0–3.0 Gy fractions) for palliation in patients with MM. Chemotherapy was administered in cases of advanced or disseminated disease according to the National Comprehensive Cancer Network Guidelines for MM^6^ based on individual patient characteristics and disease factors.

### 2.4 Follow‐up

Following the operation and initial treatments, two neurosurgeons completed the follow‐up to monitor disease progression and assess treatment response at specific time intervals. The focus of the follow‐up included evaluating post‐operative chief complaints, disease progression, recurrence, overall survival, and the management of chemotherapy and radiotherapy. Additionally, the diagnosis of MM was closely monitored during the follow‐up period. Tumor progression included tumor recurrence indicated by a notable increase in imaging findings and progression to MM, defined as bone marrow plasmacytosis ≥10% or when plasmacytoma presented with multiple lesions according to IMWG criteria. Diagnosis of disease progression involved a multidisciplinary team including hematologists, radiologists, and pathologists from our hospital. Additional follow‐ups were scheduled in cases of deteriorating patient conditions after the last contact or treatment.

### 2.5 Literature screening and selection

This study initially screened published literature from January 2000 to June 2022 including all English full‐text articles involving intracranial plasmacytomas. The search criteria were based on keywords related to intracranial plasmacytomas. The searching query = (intracranial OR clival[Title/Abstract]) OR skull base[Title/Abstract] OR dural[Title/Abstract] OR clivus[Title/Abstract] OR dura[Title/Abstract] OR cranium[Title/Abstract] OR meningeal[Title/Abstract] OR meninges[Title/Abstract] OR cranial[Title/Abstract] OR calvarial[All Fields] AND (“plasmacytoma”[MeSH Terms] OR plasmacytoma[Text Word] OR plasma cell tumor[Text Word]) AND (“2000/01/01”[PDAT]: “2022/06/01”[PDAT]). The exclusion criteria for the literature review were as follows: (1) studies without an available abstract; (2) articles written in languages other than English; (3) studies without available full text; (3) studies conducted on non‐human subjects or animal models; (4) cases with non‐plasmacytoma lesion; (5) cases with non‐intracranial location information; (6) not original case reports; and (7) studies with insufficient clinical information. Two authors reviewed each candidate article to identify and exclude duplicates based on their sources and publication dates.

### 2.6 Statistical analysis

Statistical analyses were performed on the cases from our center (*n* = 38) and prior literature (*n* = 152). Survival outcomes were summarized using the Kaplan–Meier method, and the log‐rank test was employed to assess the differences between the survival curves. Univariate and multivariate analyses were conducted using Cox's proportional hazards regression model to explore the associations between clinical and pathological characteristics and patient survival. Fisher's exact test was used for nominal variables. Hazard ratios were reported with 95% CIs. Statistical significance was defined as *p* < 0.05. All statistical tests were two‐sided. All the statistical analyses were conducted using the R programing language (version 4.1.0.) and Prism 9.0.

## 3 RESULTS

### 3.1 The diverse clinical and radiological manifestation of intracranial plasmacytoma would lead to high ratio of pre‐operative misdiagnosis

**FIGURE 1:**
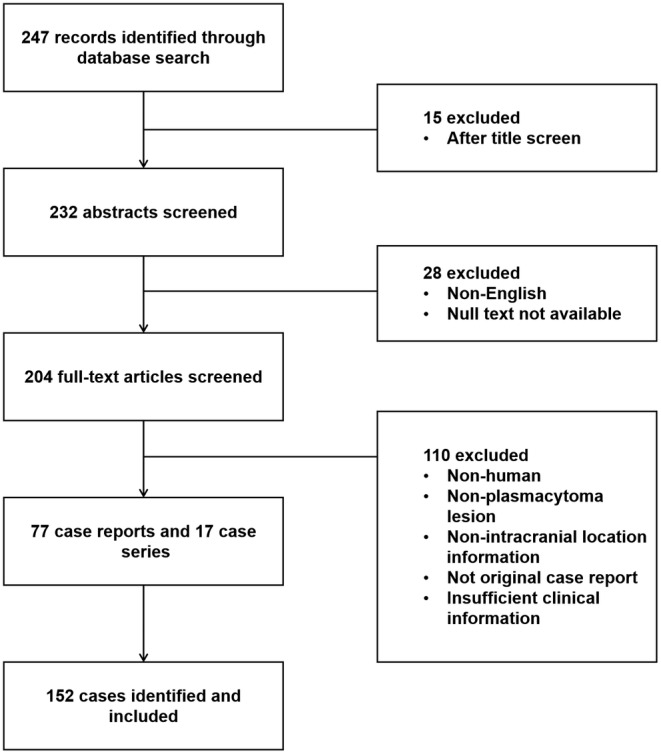
Prisma flow diagram illustrating the process of literature review. A total of 247 articles were identified through electronic database searches. After removing articles through title screening, 232 articles remained. After writing language and article retrieval methods screening, 204 articles were included for full‐text assessment. Finally, 152 cases from 77 case reports and 17 case series met the inclusion criteria and were included in this systematic review.

**FIGURE 2:**
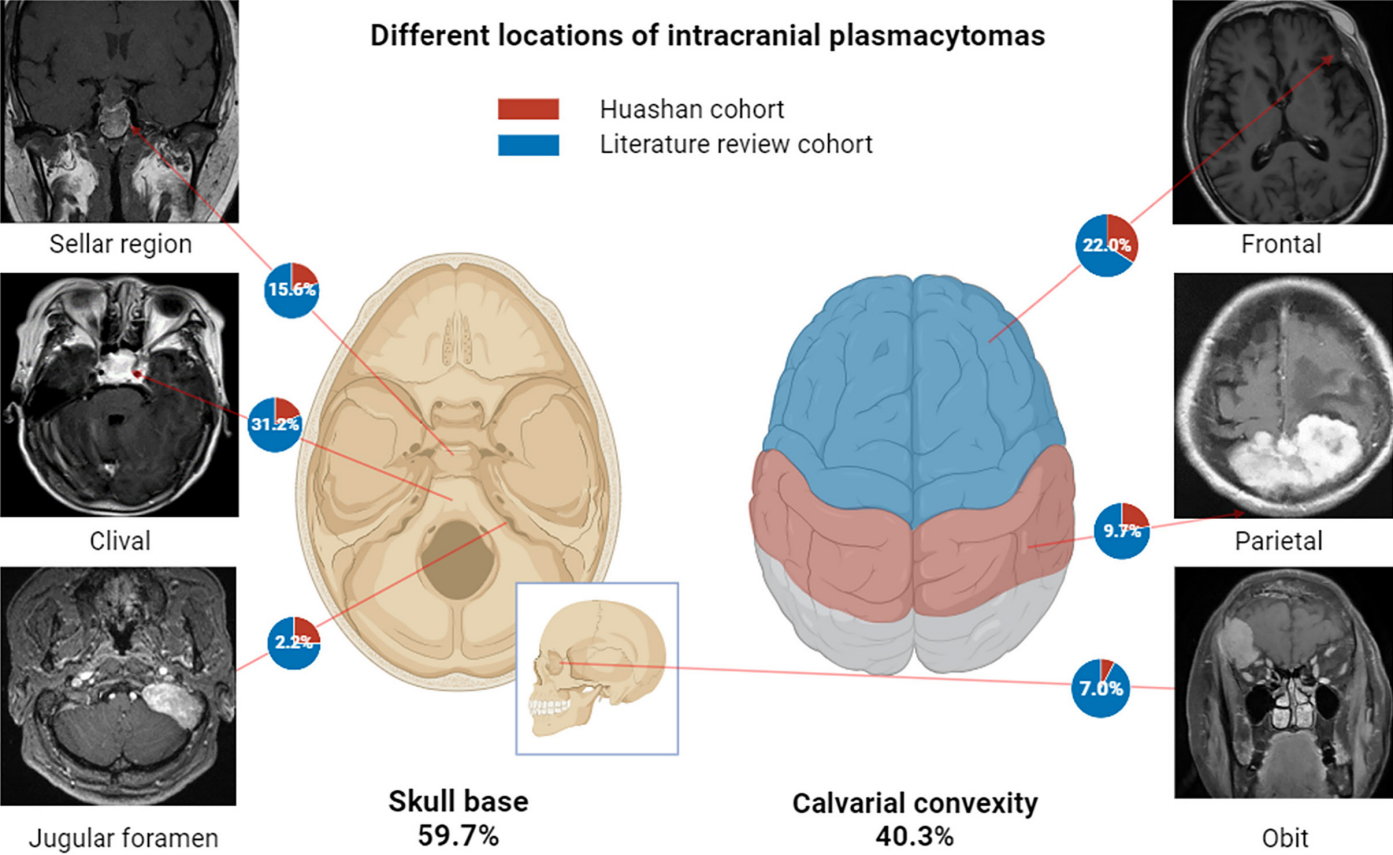
Distribution of intracranial plasmacytomas. Skull base region mainly includes the sellar region, clivus, and jugular foramen. Calvarial convexity region includes the frontal, parietal, and orbital areas. Other regions include occipital, temporal, intraventricular, nasopharyngeal areas etc. Illustrations were created in *Biorender*.

Totally, 190 patients with intracranial plasmacytoma including 38 from our center and 152 available from literature were combined for analysis, as the evaluation was described in the flow chart (Figure 1). The gender distribution is evenly balanced with 98 males and 90 females (54.1% vs. 47.9%, *p* = 0.51), and the mean age was 55.8 ± 13.1‐year‐old (range 24–84), as shown in Table 1. The most common presenting symptoms reported were headache (46.4%), oculomotor paralysis (23.2%), blurred vision (24.3%), dizziness (11.6%), facial numbness (6.6%), tinnitus and hearing loss (5.0%), vomiting (5.0%), limbs numbness (6.1%), and gait disturbance (5.0%). The specific signs observed mainly depended on the location and multiplicity of the tumors. Lesions were found in the calvaria convexity (40.3%), including frontal (36.8%), temporal (15.8%), occipital (10.5%), and parietal regions (10.5%), as well as in the skull base (59.7%) including sellar region (15.6%), petroclival (31.2%), jugular foramen (2.2%), orbit (7.0%), and other locations (5.4%) (Figure 2). It can be distributed in the left side (34.0%), right side (35.8%), and mid (30.2%). There were no significant differences observed between the Huashan cohort and the literature cohort, except for their presenting symptoms (*p* = 0.008). The Huashan cohort exhibited a higher incidence of blurred vision (34.2% vs. 21.7%, *p* = 0.11), while the literature review cohort had a greater prevalence of oculomotor nerve paralysis (10.5% vs. 26.6%, *p* = 0.037).

**TABLE 1:** Clinical characteristics of intracranial plasmacytoma.

Characteristic	Huashan cohort (*n* = 38)	Literature reviews (*n* = 152)	Integrated cohort (*n* = 190)	*p* Value
Age (years)	57.8 ± 9.6	55.2 ± 13.8	55.8 ± 13.1	0.282
Gender	(*n* = 38)	(*n* = 150)	(*n* = 188)	0.510
Male	18 (60.0%)	80 (53.3%)	98 (52.1%)	
Female	20 (40.0%)	70 (46.7%)	90 (47.9%)	
Sides	(*n* = 38)	(*n* = 121)	(*n* = 159)	0.726
Mid	11 (28.9%)	37 (30.6%)	48 (30.2%)	
Right	12 (31.6%)	45 (37.2%)	57 (35.8%)	
Left	15 (39.5%)	39 (32.2%)	54 (34.0%)	
Location	(*n* = 38)	(*n* = 148)	(*n* = 186)	0.173
Calvaria convexity	19 (50%)	56 (37.8%)	75 (40.3%)	
Frontal	14 (36.8%)	27 (18.2%)	41 (22.0%)	
Temporal	6 (15.8%)	9 (6.1%)	15 (8.0%)	
Occipital	4 (10.5%)	7 (4.7%)	11 (5.9%)	
Parietal	4 (10.5%)	14 (9.5%)	18 (9.7%)	
Skull base	19 (50%)	92 (62.2%)	111 (59.7%)	
Sellar region	6 (15.8%)	23 (15.5%)	29 (15.6%)	
Petroclival	11 (28.9%)	47 (31.8%)	58 (31.2%)	
Jugular foramen	1 (2.6%)	3 (2.0%)	4 (2.2%)	
Orbit	1 (2.6%)	12 (8.1%)	13 (7.0%)	
Others	1 (2.6%)	9 (6.1%)	10 (5.4%)	
Symptoms	(*n* = 38)	(*n* = 143)	(*n* = 181)	0.008
Headache	17 (44.7%)	67 (46.9%)	84 (46.4%)	
Oculomotor paralysis	4 (10.5%)	38 (26.6%)	42 (23.2%)	
Blurred vision	13 (34.2%)	31 (21.7%)	44 (24.3%)	
Dizziness	10 (26.3%)	11 (7.7%)	21 (11.6%)	
Facial numbness	4 (10.5%)	8 (5.6%)	12 (6.6%)	
Tinnitus and hearing loss	3 (7.9%)	6 (4.2%)	9 (5.0%)	
Vomiting	2 (5.3%)	7 (4.9%)	9 (5.0%)	
Limb weakness	0 (0%)	11 (7.7%)	11 (6.1%)	
Gait disturbance	0 (0%)	9 (6.3%)	9 (5.0%)	
Others	20 (52.6%)	105 (73.4%)	125 (69.1%)	
CT features	(*n* = 25)	(*n* = 28)	(*n* = 53)	0.17
Hyperintense	14 (56.0%)	18 (64.3%)	32 (60.4%)	
Hypointense	4 (16.0%)	2 (7.1%)	6 (11.3%)	
Isointense	2 (8.0%)	8 (28.6%)	10 (18.9%)	
Mixed intense	5 (20.0%)	0 (0.0%)	5 (9.4%)	
MRI features on T1	(*n* = 21)	(*n* = 57)	(*n* = 78)	0.10
Hypointense	9 (42.9%)	11 (29.2%)	20 (25.6%)	
Isointense	10 (47.6%)	35 (61.4%)	45 (57.7%)	
Hyperintense	2 (9.5%)	11 (29.7%)	13 (16.7%)	
MRI features on T2	(*n* = 19)	(*n* = 51)	(*n* = 70)	0.66
Hypointense	2 (10.5%)	9 (17.6%)	11 (15.7%)	
Isointense	6 (31.6%)	18 (35.3%)	24 (34.3%)	
Hyperintense	11 (57.9%)	24 (47.1%)	35 (50.0%)	
MRI enhancement	(*n* = 20)	(*n* = 73)	(*n* = 93)	0.23
Homogenously	13 (65.0%)	57 (78.1%)	70 (75.3%)	
Heterogeneously	7 (35.0%)	16 (21.9%)	23 (24.7%)	
Pre‐operation diagnosis	(*n* = 38)	(*n* = 152)	(*n* = 190)	0.068
Misdiagnosis	26 (68.4%)	79 (52.0%)	105 (55.3%)	
Unknown	12 (31.6%)	73 (48.0%)	85 (44.7%)	
Pre‐operation diagnosis (Calvarial convexity)	(*n* = 18)	(*n* = 54)	(*n* = 72)	
Meningioma	10 (55.6%)	14 (25.9%)	24 (33.3%)	
Epidural hematoma	0 (0.0%)	2 (3.7%)	2 (2.8%)	
Paraganglioma	0 (0.0%)	1 (1.9%)	1 (1.4%)	
Hemangiopericytoma	0 (0.0%)	1 (1.9%)	1 (1.4%)	
Eosinophilic granuloma	0 (0.0%)	1 (1.9%)	1 (1.4%)	
Metastatic tumor	1 (5.6%)	3 (5.6%)	4 (5.6%)	
Unknown	7 (38.9%)	32 (59.3%)	39 (54.2%)	
Pre‐operation diagnosis (Skull base except sellar)	(*n* = 14)	(*n* = 66)	(*n* = 74)	
Chordoma	4 (28.6%)	21 (31.8%)	25 (33.8%)	
Meningioma	4 (28.6%)	7 (10.6%)	11 (14.9%)	
Schwannoma	1 (7.1%)	1 (1.5%)	2 (2.7%)	
Squamous carcinoma	0 (0.0%)	1 (1.5%)	1 (1.4%)	
Paraganglioma	0 (0.0%)	1 (1.5%)	1 (1.4%)	
Unknown	5 (35.7%)	35 (53.0%)	40 (54.1%)	
Pre‐operation diagnosis (Sellar region)	(*n* = 6)	(*n* = 22)	(*n* = 28)	
Pituitary adenoma	4 (66.7%)	12 (54.5%)	16 (57.1%)	
Chordoma	2 (33.3%)	4 (18.2%)	6 (21.4%)	
Meningioma	0 (0.0%)	3 (13.6%)	3 (10.7%)	
Unknown	0 (0.0%)	3 (13.6%)	3 (10.7%)	
Intra‐operation features	(*n* = 36)	/	/	
Abundant blood supply	28 (77.8%)	/	/	
Bone invasion	32 (88.9%)	/	/	
Dura invasion	25 (69.4%)	/	/	
Hyperemic	14 (38.9%)	/	/	
Soft	15 (41.7%)	/	/	
Surgical results	(*n* = 37)	(*n* = 90)	(*n* = 127)	<0.0001
Total removal	16 (43.2%)	30 (33.3%)	46 (36.2%)	
Subtotal removal	18 (48.7%)	8 (8.9%)	26 (20.3%)	
Partial removal	0 (0.0%)	20 (22.2%)	20 (15.8%)	
Biopsy	3 (8.1%)	32 (35.6%)	35 (27.6%)	
Post‐operative diagnosis	(*n* = 35)	(*n* = 149)	(*n* = 184)	0.173
MM‐IPFP	17 (48.6%)	48 (32.2%)	65 (35.3%)	
EMP	10 (28.6%)	50 (33.6%)	60 (32.6%)	
SBP	8 (22.9%)	51 (34.2%)	59 (32.1%)	
Follow‐up	(*n* = 35)	(*n* = 120)	(*n* = 155)	
Period (Months)	54.6 (2–172)	32.9 (1–180)	42.6 (1–230)	
Patients alive	23 (65.7%)	104 (86.7%)	127 (81.9%)	
Median survival time	141 months	Not determined	141 months	0.3
Progression to MM (proportion of SIP cases)	5 (27.8%)	19 (21.3%)	24 (22.4%)	0.64
Post‐operative treatment	(*n* = 38)	(*n* = 151)	(*n* = 189)	
RT alone	3 (7.9%)	55 (36.4%)	58 (30.7%)	
CMT alone	12 (31.6%)	22 (14.6%)	34 (18.0%)	
RT + CMT	10 (26.3%)	50 (33.1%)	60 (31.7%)	
Neither	10 (26.3%)	10 (6.6%)	20 (10.6%)	
Unknown	3 (7.9%)	14 (9.3%)	17 (9.0%)	
Pathology				
CD138	(*n* = 35)	(*n* = 55)	(*n* = 90)	
(+)	35 (100%)	44 (100%)	90 (100.0%)	
Ki‐67	23.4 ± 22.6% (1%–90%)	15.9 ± 15.9% (1%–60%)	19.9 ± 20.1% (1%–90%)	
Light chain IHC	(*n* = 39)	(*n* = 102)	(*n* = 141)	
Kappa (+)	28 (71.8%)	64 (62.7%)	73 (68.2%)	
Lambda (+)	22 (56.4%)	36 (35.3%)	46 (43.0%)	

Abbreviations: CT, chemotherapy; CT, computed tomography; MM, multiple myeloma; MRI, magnetic resonance imaging; RT, radiotherapy.

Various imaging features resulted in a high misdiagnosis ratio of intracranial plasmacytoma. CT scans revealed diverse features, including bone erosion, but failed to provide a definitive diagnosis due to variations in signal intensity, which presented as hyperintense (60.4%), hypointense (11.3%), isointense (18.9%), or mixed intense (9.4%). On T1‐weighted MR images, the most common signal intensity was isointense (57.7%), followed by hypointense (25.6%) and hyperintense (16.7%). On T2‐weighted MR images, the signal intensity appeared as hyperintense (50.0%), hypointense (15.7%), or isointense (34.3%). MR enhancement was non‐specific, with findings of homogenously (75.3%) or heterogeneously (24.7%). Additional radiological examinations, including CTA, MRV, DTI, and fMRI, were performed in some cases, but no specific radiological features were observed.

As intracranial plasmacytoma mimics other intracranial lesion clinically and radiologically, pre‐operation diagnosis remains challenging. The most common misdiagnoses included meningioma, chordoma, and pituitary adenoma, accounting for 33.3% (*n* = 24) of cases in the calvarial convexity, 33.8% (*n* = 25) on the skull base, and 57.1% (*n* = 16) in the sellar region, respectively. Other misdiagnoses such as epidural hematoma (2.8%), paraganglioma (1.4%), eosinophilic granuloma (1.4%), hemangiopericytoma (1.4%) and metastases (5.6%), were found in nine patients with calvaria convexity involvement. Chordoma (21.4%) and meningioma (10.7%) were frequently misdiagnosed in patients with plasmacytomas in the sellar region. The high rate of misdiagnosis complicates timely interventions and underscores the need for precise optimization based on further preoperative and intraoperative findings.

### 3.2 Neurosurgical management and follow up

Among the 127 patients with documented surgical outcomes, the operation performed consisted of total removal (46.2%), subtotal removal (20.3%), partial removal (15.8%), and biopsy (27.6%). Notably, a significant difference was observed between the Huashan cohort and the literature review cohort, as shown in Table. 1. The choice of surgical approach mainly depended on tumor location and included microsurgery and endoscopic surgery. In the Huashan cohort, one patient with a left maxillary sinus lesion underwent left paranasal sinus biopsy. Among the 20 craniotomies, seven patients had their bone flap reset, while 13 patients had their bone flap discarded. Of these, 11 patients underwent one‐stage operation with titanium plate shaping. For the 12 trans‐sphenoid surgeries, none of them mentioned cerebrospinal fluid leakage. Notably, one case in the sellar region experienced excessive intraoperative blood loss and symptoms of shock, prompting the surgeons to pause the operation and control the blood pressure. The most common gross characteristics of intracranial plasmacytoma were hyperemic (38.9%), bone invasion (88.9%), and dura invasion (69.4%). Following postoperative bone marrow biopsy and comprehensive systemic assessment, 65 patients (35.3%) were identified as having MM, presenting initially with intracranial plasmacytoma (MM‐IPFP). Sixty cases (32.6%) were diagnosed as EMP, while 59 cases (32.1%) were classified as SBP. Pathological results showed a wide distribution of Ki‐67 values ranging from 1% to 90%, with a median of 19.9 ± 20.1% (n = 72). Immunohistochemistry showed that all tumors (*n* = 90) were positive for CD138. Monoclonal kappa and lambda light chain expression were identified in 73 (68.2%) and 46 (43.0%) patients, respectively. In summary, patients with intracranial plasmacytoma lack specific clinical features at presentation and during intraoperative evaluation.

Follow‐up data were available for 155 cases, with a mean follow‐up period of 42.6 months (range 1–230). At the time of the latest follow‐up evaluation, 127 (81.9%) patients were still alive, with a median survival of 141 months. There was no significant difference between the Huashan cohort and the literature review cohort (141 months vs. not determined, *p* = 0.3). Until the last follow‐up, 24 (22.4%) patients with SIP progressed to MM. Combined RT and CMT were used in 60 (31.7%) patients. Additionally, 58 (30.7%) patients and 34 (18.0%) patients received RT alone and CMT alone, respectively, while 20 (10.6%) patients did not receive any form of treatment.

### 3.3 Radiotherapy not surgery would improve the prognosis of MM‐IPFP

**FIGURE 3:**
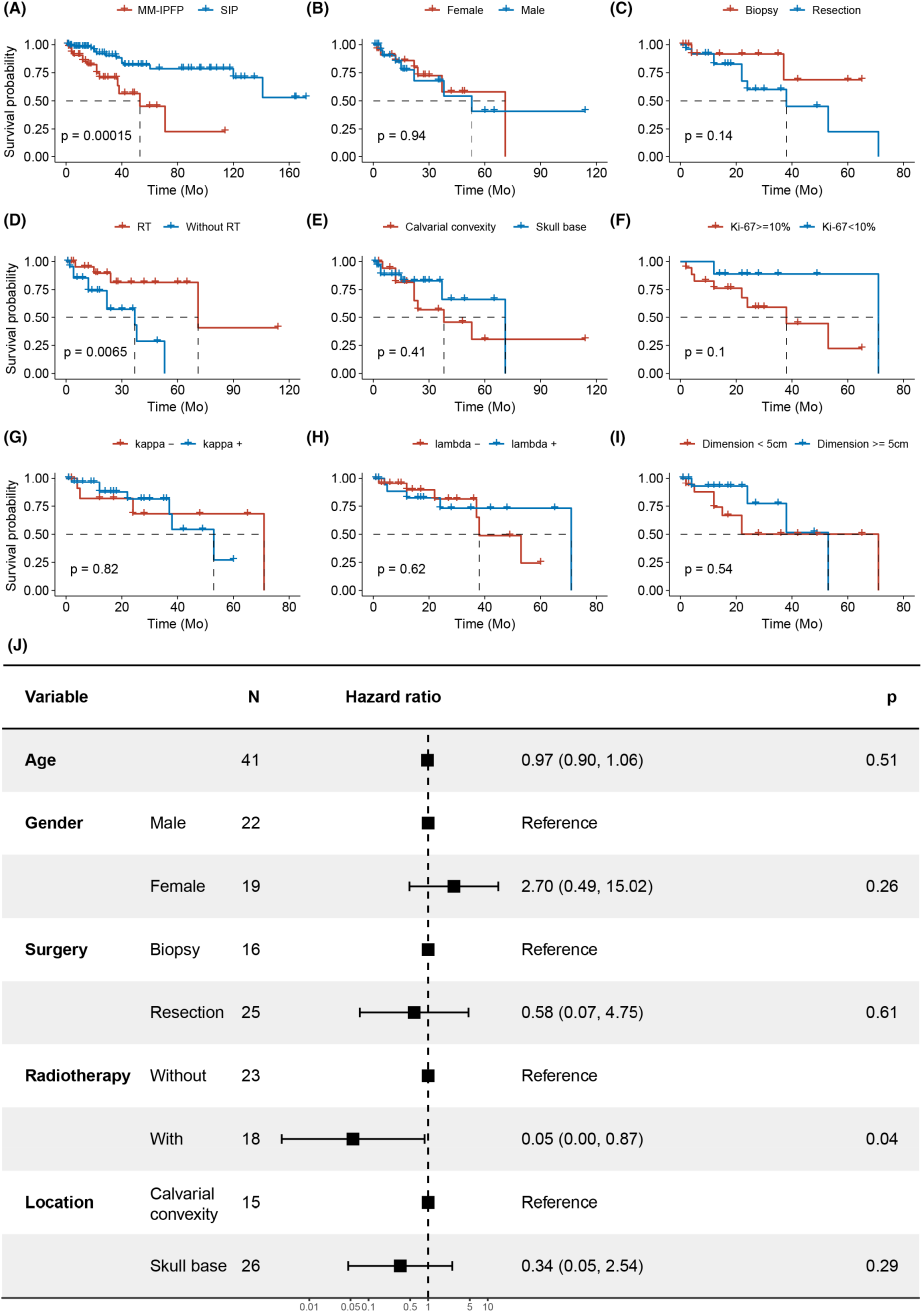
Survival analysis and multivariable analysis of the prognostic factors of MM‐IPFP. (A) Kaplan–Meier survival curves of patients with MM‐IPFP and SIP. (B–I) Kaplan–Meier survival curves of patients stratified by gender (B), surgical options (C), post‐operative RT (D), tumor location (E), Ki‐67 index (F), light chain staining (G and H) and dimensions of intracranial lesions (I) in MM‐IPFP. (J) The multivariate Cox analysis for MM‐IPFP.

**FIGURE 4:**
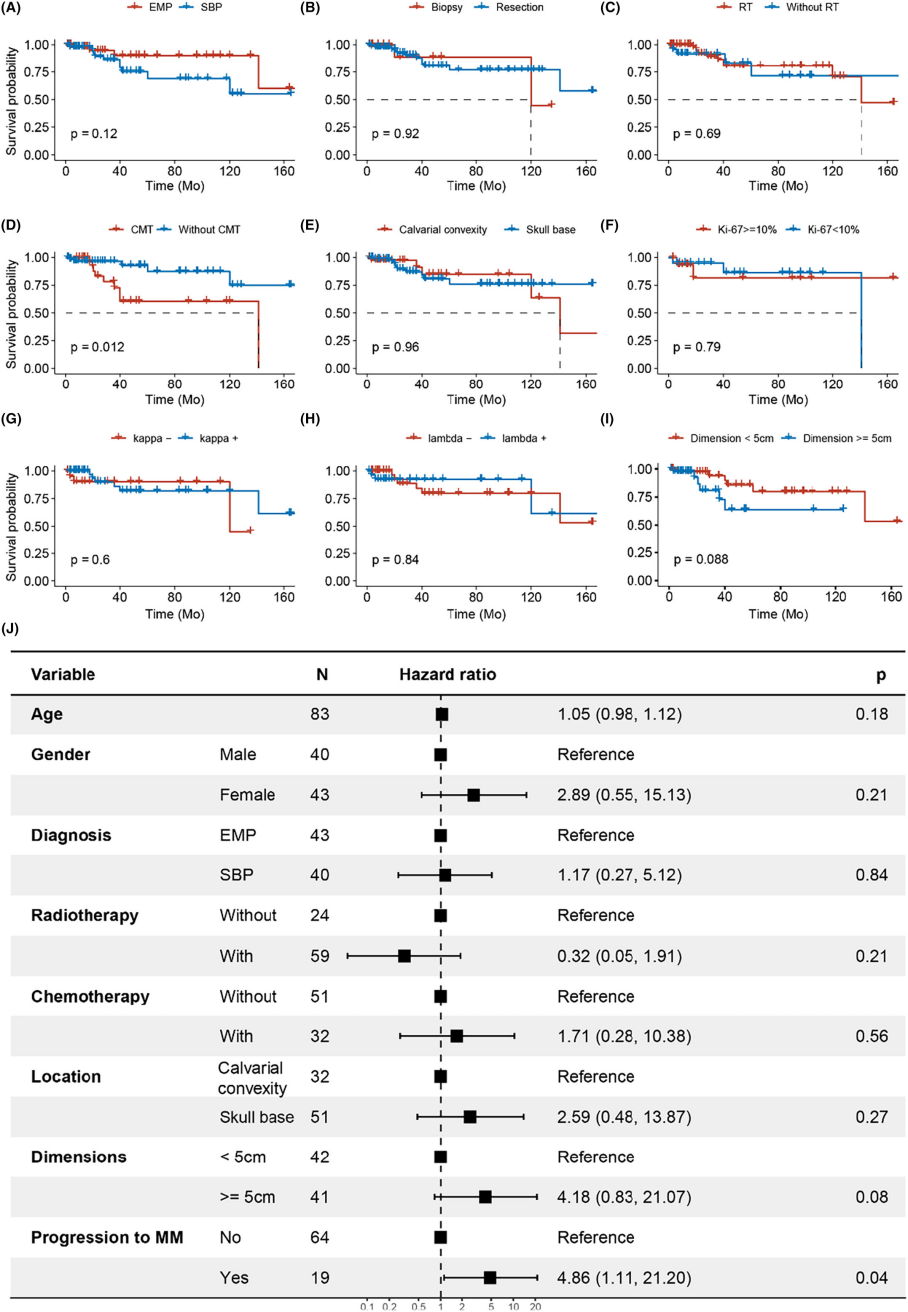
Survival analysis and multivariable analysis of the prognostic factors of SIP. (A) Kaplan–Meier survival curves of all patients with SBP and EMP. (B–I) Kaplan–Meier survival curves of patients stratified by surgical options (B), post‐operative treatments (C and D), tumor location (E), Ki‐67 index (F), light chain staining (G and H) and dimensions of intracranial lesions (I) in SIP. (J) The multivariate Cox analysis for SIP.

To investigate the prognostic value of clinicopathological features in intracranial plasmacytomas, survival and Cox analysis were conducted on a combined cohort from our center and literature reviews. A total of 155 patients with an average follow‐up period of 41.7 months were included. SIP demonstrated significantly improved survival compared to MM‐IPFP by Kaplan–Meier curves (SIP vs. MM‐IPFP, mean time not determined vs. 53 months, *n* = 107 vs. 48, *p* < 0.05, Figure 3A). Separate analyses were performed for the MM‐IPFP (Figure 3) and SIP (Figure 4) groups, evaluating general patient conditions, surgery outcomes, tumor dimensions and locations, pathological features, and the effects of post‐operation management on survival. For the MM‐IPFP group, univariate Cox analysis revealed that only RT (HR, 0.193; 95% CI, 0.052–0.712; *p* = 0.0135) was a risk factor for the survival (Table. 2). In the SIP group, univariate Cox analysis identified age (HR, 1.07; 95% CI, 1.01–1.13; *p* = 0.0195) and CMT (HR, 3.76; 95% CI, 1.25–11.3; *p* = 0.0188) as risk factors for survival (Table. 3). Tumor dimension (HR, 2.79; 95% CI, 0.823–9.48; *p* = 0.0995) was marginally associated with survival. Furthermore, we investigated the effects of clinicopathologic factors on survival.

**TABLE 2:** The univariate Cox analysis results for MM‐IPFP group.

Variable		HR	Lower_95	Upper_95	*p* Value
Age	Increasing	1.02	0.972	1.070	0.429
Gender	“Female” versus “male”	0.959	0.329	2.8	0.938
Location	“Skull base” versus “Calvarial convexity”	0.638	0.22	1.85	0.408
Dimension	“≥5 cm” versus “<5 cm”	0.67	0.195	2.3	0.524
Surgery	“Resection” versus “Biopsy”	3.05	0.654	14.3	0.156
Radiotherapy	“With” versus “without”	0.193	0.0523	0.712	**0.0135**
Ki‐67	“≥10%” versus “<10%”	4.73	0.59	37.8	0.143
Light chain κ	“+” versus “−”	1.18	0.302	4.61	0.812
Light chain λ	“+” versus “−”	0.73	0.206	2.59	0.626

The bold values in the '*p*‐value' column represented data points that have reached statistical significance.

**TABLE 3:** The univariate Cox analysis results for SIP group.

Variable		HR	lower_95	upper_95	*p* Value
Age	Increasing	1.07	1.01	1.13	**0.0195**
Gender	“Female” versus “male”	2.28	0.71	7.35	0.166
Location	“Skull base” versus “Calvarial convexity”	1.01	0.337	3.04	0.983
Dimension	“≥5 cm” versus “<5 cm”	2.79	0.823	9.48	0.0995
Surgery	“Resection” versus “Biopsy”	0.756	0.166	3.43	0.717
Radiotherapy	“With” versus “without”	0.79	0.246	2.54	0.692
Chemotherapy	“With” versus “without”	3.76	1.25	11.3	**0.0188**
Ki‐67	“≥10%” versus “<10%”	1.36	0.223	8.28	0.74
Light chain κ	“+” versus “−”	0.587	0.139	2.47	0.468
Light chain λ	“+” versus “−”	0.952	0.237	3.82	0.945
Progression to MM	“progression” versus “no progression”	5.43	1.81	16.3	**0.00252**

The bold values in the '*p*‐value' column represented data points that have reached statistical significance.

The effects of clinicopathological factors on survival were further investigated. For the MM‐IPFP group, the Kaplan Meier Curve revealed that patients who received post‐operation RT had significantly longer survival compared to those who did not (with RT vs. without RT, median survival 71 months vs. 37 months, *n* = 25 vs. 23, *p* < 0.05, Figure 3D). While in the SIP group, patients who underwent post‐operation CMT had a poorer prognosis compared to those without CMT (*n* = 38 vs. 64, *p* < 0.05, Figure 4D). Although not statistically significant, a trend towards prolonged survival was observed with a low Ki‐67 index in the MM‐IPFP group (Ki67 < 10% vs. Ki67 ≥ 10%, median survival 72 vs. 38 months, *n* = 7 vs. 21 *p* = 0.1; Figure 3C) and with smaller tumor dimensions in the SIP group (dimension<5 cm vs. dimension≥5 cm, *n* = 42 vs. 41 *p* = 0.088; Figure 4I). Gender, surgical strategy, tumor locations, and light chain staining did not show a significant difference in survival for both the MM‐IPFP and SP groups (Figure 3B–I and Figure 4A–I). Further multivariate analysis indicated that only RT (HR, 0.05; 95% CI, 0.00–0.87; *p* = 0.04, Figure 3J) was significantly associated with positive prognosis in MM‐IPFP, while progression to MM (HR, 4.86; 95% CI, 1.11–21.20; *p* = 0.04, Figure 4J) was significantly associated with poor prognosis in SIP.

### 3.4 Ki‐67 index could predict the progression of SIP to MM

**FIGURE 5:**
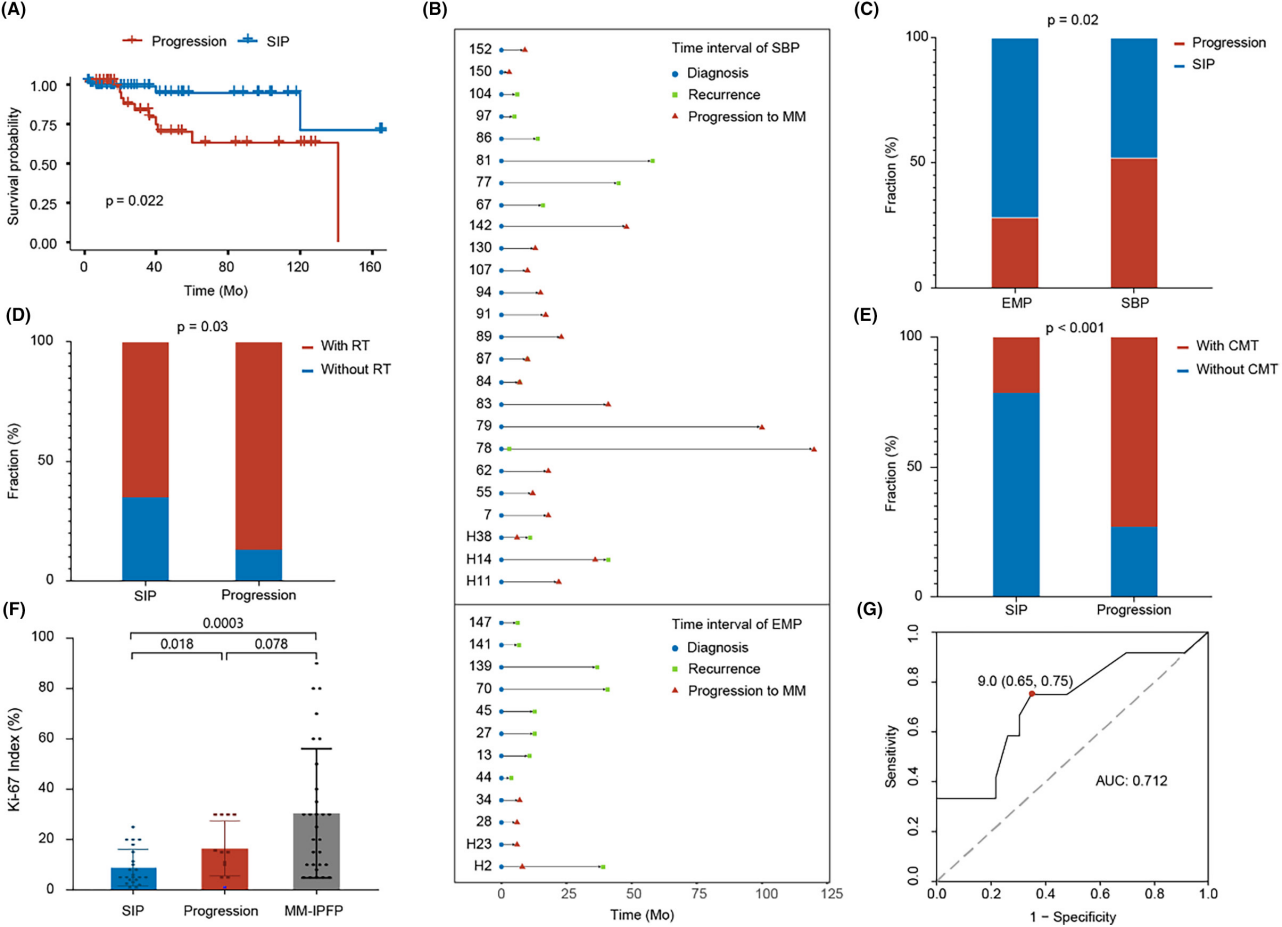
Clinicopathological differences between SIP with or without post‐therapeutic progression. (A) Kaplan–Meier survival curves of SIPs with or without progression to MM. (B) Time interval of SBP and EMP progression. Blue circle points represent the time points of diagnosis. Green squares represent the time points of recurrence. Red triangles represent the time points of progression to MM. (C) Histogram demonstrating the fraction of patients with EMP and SBP progressing to MM (Fisher's exact test *p*‐value = 0.02). (D) Histogram demonstrates the fraction of SIP patients with or without progression to MM receiving post‐operative RT (Fisher's exact test *p*‐value = 0.03). (E) Histogram demonstrates the fraction of SIP patients with or without progression to MM receiving post‐operation CMT (Fisher's exact test *p*‐value < 0.001). (F) Comparison of Ki‐67 index between SIP, SIP with progression (including progression to MM and recurrence) and MM‐IPFP. Unpaired *t*‐test was used to calculate the *p*‐values (SIP vs. progression, *p*‐value = 0.018; SIP vs. MM‐IPFP, *p*‐value = 0.0003; progression vs. MM‐IPFP, *p*‐value = 0.078) (G) ROC curve for Ki‐67 indexes in predicting SIP progression into MM. AUC = 0.712 with cutoff value of 9.0%.

Progression to MM and recurrence are milestone events in the progression of SIP. We included 94 patients with complete follow‐up of more than 6 months after surgery for analysis (Table. 4) and divided them into the SIP with or without progression groups based on the IMWG criteria.^2^ There is a significant difference in survival between the progression group and the SIP group (progression vs. SIP, median survival 141 months vs. not determined, *n* = 36 vs. 56, *p* = 0.022; Figure 4A). Further, we found that SBP had a similar time interval of progression (SBP vs. EMP, mean time 22.2 months vs. 12.7 months, *n* = 25 vs. 13, *p* = 0.17, Figure 5B) and a significantly higher proportion of progression (SBP vs. EMP, *n* = 52.1% vs. 28.3%, *p* = 0.02, Figure 5C) compared to EMP. The impacts of progression were further investigated through a comparative analysis between the two groups. A significant correlation between post‐operative treatment and progression was observed, as the progression group had a significant higher percentage of patients receiving RT (progression vs. SIP, 86.8% vs. 64.8%, *n* = 38 vs. 54, *p* = 0.03, Figure 5D) and CMT (progression vs. NPSIP, 73.0% vs. 21.2%, *n* = 37 vs. 52, *p* < 0.0001, Figure 5E) compared to the SIP group. Among the other factors explored, only the pathology Ki‐67 index showed a significant difference (*p* = 0.018, Figure 5F). The mean Ki‐67 value of SIP group was 8.82% ± 7.03 (*n* = 23, range 1%–25%), while the mean Ki‐67 value of progression group was 16.5% ± 10.5 (*n* = 12, range 1–30%). Using ROC curve analysis, we determined that a Ki‐67 index of 9.0% was the optimal cutoff value for predicting progression of SIP, with an area under the curve of 0.712 (Figure 5G). Additionally, there was a slight predominance of older age (57.2 ± 10.7 vs. 52.9 ± 13.7 years, progression vs. SIP, *n* = 38 vs. 56, *p* = 0.11), a higher percentage of females (62.2% vs. 41.1% female percentage, progression vs. SIP, *n* = 38 vs. 56, *p* = 0.15) and greater tumor dimension (58.1% vs. 53.8% ≥5 cm percentage, progression vs. SIP, *n* = 31 vs. 42, *p* = 0.10) in the progression group. However, no significant differences were observed in surgical outcomes (84.2% vs. 88.9% resection percentage, progression vs. SIP, *n* = 38 vs. 54, *p* = 0.54) or tumor location (71.1% vs. 58.9% skull base percentage, progression vs. SIP, *n* = 38 vs. 56, *p* = 0.28) between the two groups.

**TABLE 4:** Clinicopathological features between SIPs with or without post‐therapeutic progression.

Characteristic	Progression Group (*n* = 38)	SIP Group (*n* = 56)	*p* Value
Age (years)	57.2 ± 10.7	52.9 ± 13.7	0.11
Gender	(*n* = 37)	(*n* = 56)	0.15
Male	14 (37.8%)	30 (53.6%)	
Female	23 (62.2%)	26 (46.4%)	
Initial diagnosis	(*n* = 38)	(*n* = 56)	0.02
EMP	13 (34.2%)	33 (58.9%)	
SBP	25 (65.8%)	23 (41.1%)	
Location	(*n* = 38)	(*n* = 56)	0.28
Calvarial convexity	11 (28.9%)	23 (41.1%)	
Skull base	27 (71.1%)	33 (58.9%)	
Dimensions	(*n* = 31)	(*n* = 42)	0.10
<5 cm	13 (41.9%)	26 (46.2%)	
≥5 cm	18 (58.1%)	16 (53.8%)	
Surgery	(*n* = 38)	(*n* = 54)	0.54
Biopsy	6 (15.8%)	6 (11.1%)	
Resection	32 (84.2%)	48 (88.9%)	
Radiotherapy	(*n* = 38)	(*n* = 54)	0.03
With	33 (86.8%)	35 (64.8%)	
Without	5 (13.2%)	19 (35.2%)	
Chemotherapy	(*n* = 37)	(*n* = 52)	<0.0001
With	27 (73.0%)	11 (21.2%)	
Without	10 (27.0%)	41 (78.8%)	
Ki‐67 index	(*n* = 12)	(*n* = 23)	0.019
Mean	16.5% ± 10.5	8.82% ± 7.03	
Follow‐up	(*n* = 36)	(*n* = 56)	0.92
Period (Months)	50.9 (7–230)	53.19 (7–172)	
Median survival time	141 months	Not determined	<0.05
Time interval of MM progression (Months)	23.3 (3–120)	/	
Time interval of recurrence (Months)	19.8 (3–58)	/	

## 4 DISCUSSION

Plasmacytomas are characterized by the abnormal proliferation of clonal, immunoglobulin‐secreting plasma cells. Typically, they manifest as localized collections of plasma tumor cells and rarely involve intracranial regions.^1^ Through a retrospective analysis of 190 cases, we found that intracranial plasmacytomas tend to occur with high frequency on the calvaria convexity, particularly the frontal lobe and skull base regions. Long‐term survival is contingent upon post‐operative management of RT for MM‐IPFP. Progression to MM is a poor prognostic factor for SIP, and Ki‐67 index shows promise as a candidate for predicting the possibility of progression from SIP to MM.

Intracranial plasmacytomas often present as lesions with bone erosion and an abundant blood supply, which can be revealed through imaging techniques such as CT bone window and MRI enhancement and further confirmed by intra‐operation situation and pathology.^4^ However, preoperative diagnosis of intracranial plasmacytomas remains challenging due to the lack of obvious radiological characteristics, which can hinder treatment planning and precise sampling. For lesions located on the calvaria convexity, the most common misdiagnosis was meningioma because the base of plasmacytoma located on dural may be mistaken for dural tail sign. Two features can help distinguish between plasmacytoma and meningioma. Firstly, plasmacytomas can penetrate the skull bone and invade the galea aponeurotica. Secondly, plasmacytomas usually lack calcification compared to meningiomas. In the sellar region, plasmacytomas are frequently misdiagnosed as pituitary adenomas due to the similar radiology and symptoms related to vision. However, plasmacytomas tend to cause earlier bone erosion and neurology deficits, exhibiting fewer endocrine symptoms compared to pituitary adenomas. For plasmacytomas in the skull base, the most common misdiagnosis includes chordomas, followed by meningiomas, paragangliomas, metastasis, and inflammatory conditions. Histopathological examination including CD138 positivity and immunoglobulin light chain is required for a definitive diagnosis.

In cases where plasmacytomas are misdiagnosed as other intracranial lesion preoperatively, surgery can provide the pathological confirmation and symptom relief. In our series, surgical treatment was common with a total removal rate 43.2%. The biopsy rate was lower at 8.1%, compared with 35.6% reported in literature reviews. This discrepancy may be partially attributed to the challenges in preoperative diagnosis. Survival analysis revealed no significant difference between groups with surgical strategies, which may be due in part to the high sensitivity of plasmacytomas to RT. Previous studies identified that local control rates of 80–100% can be achieved by RT, making it the treatment of choice for preventing progression to MM and recurrence.^13^ The recommended clinical radiation dose ranged between 3000 cGy and 5400 cGy.^14^ CMT regimens for plasmacytomas include the combination of lenalidomide, bortezomib, and dexamethasone for standard‐risk patients and carfilzomib, lenalidomide, and dexamethasone for high‐risk patients, followed by a proteasome‐inhibitor‐based regimen for maintenance therapy. Other regimens, including cyclophosphamide or thalidomide, are used in patients with acute renal failure.^15^ However, the impact of CMT in the treatment of solitary plasmacytoma remains a contentious topic with conflicting findings in the literature. Similarly to the study by Ma et al.,^4^ we found that SIP patients who received CMT had significantly shorter survival compared to those without CMT. However, upon conducting multivariate analysis, we observed that CMT was not an independent prognostic factor, and progression to MM may play a more significant role in contributing to the poorer prognosis. Mignot et al. reported improved outcomes when using CMT with concurrent lenalidomide‐dexamethasone compared to radiation therapy alone.^16^ Furthermore, other studies have suggested that chemotherapy is associated with a decreased risk of disease progression.^9^ Recently, autologous stem cell transplantation (ASCT) has emerged as a significant treatment option for patients with MM. Studies have showed ASCT can significantly prolong progression‐free survival,^17^ and improves median survival time by approximately 1 year.^2^ For patients with initial plasmacytoma, ASCT is also recommended for eligible transplant patients and should be taken into consideration at the first relapse.^2^, ^18^

Progression to MM is a milestone event of intracranial plasmacytomas. Previous studies have reported a 5‐year disease progression‐free survival was 52.9%.^4^ Our investigation revealed that in a 3.3‐year follow‐up period, 9.3% patients with EMP and 35.8% patients with SBP progressed to MM. We also found a tremendous difference in post‐operative survival between patients with SIP who experienced progression and those who did not. However, the clinical diagnosis of MM may not accurately reflect the true time of progression. Many patients diagnosed with MM postoperatively may already have preexisting systemic diseases at an early stage. Therefore, identification of plasmacytoma subgroup with the MM progression tendency is necessary. Through our investigation of common markers used in plasmacytomas IHC staining including CD138, light chain kappa and lambda and Ki‐67 index, we found that Ki‐67 index, with a cutoff value of 9%, can help predict the possibility of MM progression. The post‐operation classification of intracranial plasmacytoma can be based molecular pathology, where Ki‐67 index serves as a suitable candidate. Considering the significant decrease in prognosis associated with MM, a systematic examination in neurosurgery and hematology departments at least every 6 months to guarantee the immediate recognition of recurrence and MM is recommended. Strict intracranial and general examination including regular CT or MRI scan, whole‐body bone scan, bone marrow examination, serum protein electrophoresis, and B‐J protein in urine are also necessary.

The limited understanding of the molecular mechanisms underlying intracranial plasmacytomas highlights the need for further research. We compiled the existing literature on molecular events related to intracranial plasmacytomas (Table 5) and identified a case of EMP in which 13q14 deletion and *p53* deletion were detected through fluorescence in situ hybridization.^19^ This patient experienced disease progression to MM 1 year after surgery confirmed by the bone marrow biopsy. Additionally, in 5 cases of MM‐IPFP, cytogenetic abnormalities including 1q gain, 17p deletion, monosomy 13 and 14, t(11;14) and t(11;14) were detected, which have been recognized as high‐risk events of MM in the updated IMWG criteria.^2^ Recent studies have also identified genetic alterations in *KRAS*, *NRAS*, and *BRAF* that substantially contribute to the progression of EMP, presenting potential avenues for targeted therapeutic interventions.^20^ Otherwise, the acquisition of hyperdiploidy or translocations involving immunoglobulin heavy chain gene loci are initiating events driving the development of MM, widely presents in the precursor states of monoclonal gammopathy of undetermined significance and smoldering MM. As precursor states progress to MM, the frequency of genetic abnormalities including copy number variations, secondary translocations, and somatic mutations increases, leading to dysregulation of cell cycle control and subsequent disease progression.^21^ Whether tumor cells undergo clonal selection under treatment‐induced or environmental stress during the progression from intracranial plasmacytoma to MM needs further research.

**TABLE 5:** Genetic factors of patients with intracranial plasmacytoma.

Patient ID	Author	Age	Sex	Initial diagnosis	Progression (Months)	Treatment	Status	OS (Months)	Genetic factors	Methods (sample)
152	Alafaci et al.^19^	65	M	EMP	MM (9)	RT, CMT	Alive	12	13q14 deletion; p53 deletion	FISH (tumor)
68	Ibekwe et al.^22^	53	F	MM	/	RT, CMT	Alive	3	CCND1/IGH gene fusion; 1q gain	FISH (bone marrow cells)
96	Yazdanpanah et al.^23^	45	M	MM	/	RT, CMT	Alive	NA	Monosomy 13 and 14; 17p13 deletion; IGH/14q32 gene rearrangement; 1q21.3/CKS1B, 9, 15 gain	FISH (bone marrow cells)
101	Guo et al.^24^	61	F	MM	/	CMT	Alive	NA	13q14 deletion; t(11; 14) (q13; q32)	FISH (bone marrow cells)
119	Lee et al.^25^	24	M	EMP	No	RT	Alive	24	Inv (9) (p13q21)	FISH (bone marrow cells)
145	Waqar et al.^26^	60	M	MM	/	RT, CMT	Alive	1	t(4; 14)	FISH (bone marrow cells)

Abbreviations: CMT, chemotherapy; EMP, extramedullary plasmacytoma; F, female; FISH, fluorescence in situ hybridization; M, male; MM, multiple myeloma; OS, overall survival; RT, radiotherapy; SBP, solitary plasmacytoma.

## 5 CONCLUSION

Intracranial plasmacytoma is an exceedingly rare lesion that poses a diagnostic challenge prior to surgery. The prognosis of intracranial plasmacytoma is generally favorable, unless there is progression leading to the development of MM. These tumors occur with high frequency on calvaria convexity, especially in the frontal region and skull base, including the sellar region. We found that long‐term survival is conditional on post‐operative management of RT and progression to MM. The precise role of aggressive tumor resection and CMT remains uncertain. Ki‐67 index is a candidate for predicting the possibility of progression to MM. Future researches could endeavor to leverage advanced molecular biology techniques to promptly identify these high‐risk individuals at diagnosis. Consequently, it is recommended to implement rigorous intracranial and general examinations within both neurosurgery and hematology departments, aiming to proactively impede the progression of intracranial plasmacytoma. It would provide some evidence for risk factors of intracranial plasmacytoma from this large cohort.

## AUTHOR CONTRIBUTIONS

**Yuan Feng:** Data curation (equal); formal analysis (equal); investigation (equal); visualization (equal); writing – original draft (equal). **Zongpu Zhang:** Data curation (equal); formal analysis (equal); investigation (equal); supervision (equal); visualization (equal); writing – original draft (equal). **Fufang Qiu:** Data curation (equal); formal analysis (equal); investigation (equal); methodology (equal); supervision (equal); visualization (equal). **Zixiao Yang:** Data curation (equal); formal analysis (equal); investigation (equal); supervision (equal); validation (equal); visualization (equal). **Ji Xiong:** Data curation (equal); investigation (equal); resources (equal); supervision (equal). **Wei Zhu:** Data curation (equal); investigation (equal); methodology (equal); supervision (equal); validation (equal). **Fangzhu Wan:** Investigation (equal); methodology (equal); supervision (equal); visualization (equal). **Yi Zhang:** Conceptualization (equal); investigation (equal); methodology (equal); project administration (equal); supervision (equal); writing – review and editing (equal). **Wei Hua:** Conceptualization (lead); funding acquisition (lead); methodology (lead); project administration (lead); writing – review and editing (lead). **Bobin Chen:** Data curation (equal); writing – original draft (equal); writing – review and editing (equal). **Jiguang Wang:** Data curation (equal); writing – original draft (equal); writing – review and editing (equal).

## FUNDING INFORMATION

The study was funded by the National Natural Science Foundation of China (82072784 and 82103690) and the CAMS Innovation Fund for Medical Sciences (2022‐I2M‐C&T‐B‐112).

## CONFLICT OF INTEREST STATEMENT

All the authors declare that they have no conflict of interest.

## ETHICS STATEMENT

All procedures performed in studies involving human participants were approved by the institutional review board of Huashan hospital, Fudan University.

## CONSENT FOR PUBLICATION

Informed consent was obtained from all individual participants included in the study.

## Supporting information


Data S1.

Figure S1.

Table S1.

## Data Availability

The supporting data are available from the corresponding author on reasonable request.
