# Supplementary material for: Enhanced clinical outcomes with radiotherapy in diagnostically challenging intracranial plasmacytomas: Analysis of 190 cases
Source: Cancer Med. 2024 Mar 8;13(4):e7017. doi: 10.1002/cam4.7017 (PMC10922021; doi:10.1002/cam4.7017)
Supplement: Supplementary file 1 — Data S1. [file CAM4-13-e7017-s001.docx]

**Case illustrations**

**Case 1 Petroclival plasmacytoma**

A 64-year-old female suffered from headache and dizziness for 3 months was admitted in 2015. Her neurological exam was significant for cerebellar signs, as well as a positive Rhomberg with the patient falling to the left. Besides, the patient presented with decreasing blurred vision. CT revealed an equal signal petroclival tumor with bone invasion. MRI demonstrated the lesion with low T1, equal high T2 and obvious enhancement and extensions through sellar region and invasion of clival skull (Supplementary fig.1). The pre-operation diagnosis was considered petroclival chordoma. The patient received transnasal transsphenoidal endoscopic tumorectomy. After removing the bone of sphenoid sinus and clival, the hyperemic and soft tumor with abundant blood supply was revealed. The tumor was further nearly total removed piece by piece. The patient was stable with no postoperative complications and new symptoms or signs. Pathology presented 1.0*0.5*0.5cm tissue with CgA (-), ACTH (-), FSH (-), GH (-), LH (-), PRL (-), TSH (-), MIB-1 (30%), Syn (-), CD138 (+), EMA (+), κ (+), λ (-), HMB45 (-) and CK (-). Pathology diagnosis was petroclival plasmacytoma with invasion of bone.

Based on a follow-up of 1.86 year, the patient presented intermittent headache and blurred vision with no influence on social life. Post-operative examination revealed multiple myeloma. The patient received radiotherapy. Post-operation examination demonstrated recurrence in Dec 2015 but no metastasis and the patient was stable.

**Case 2 Sellar plasmacytoma**

A 47-year-old female presented with a 2-year history of headache with menopause. After exclusion of gynecological disease, she was admitted in Jun 2016 to neurosurgery department with CT revealing a sellar lesion (Supplementary fig.2). The patient denied obesity, polyuria, seizure or vomiting. Examination revealed no blurred vision or any vision defect. Blood test reviewed TSH 151.2 mIU/L, TT3 1.60nmol/L, TT4 104.6nmol/L, FT3 3.71pmol/L, FT4 11.86pmol/L; GH 0mU/L, E2 18.4, LH 1.99, FSH 5.69, PRL 24.96ng/mL, COR 3.85, Testo 0.09nmol/L, ACTH 25.80pg/ml. MRI revealed a sellar lesion with equal T1, equal T2 and obvious enhancement. The pre-operation diagnosis was pituitary adenoma.

The tumor was removed by trans-single nostril transsphenoidal microsurgery. The tumor was revealed behind the pituitary presenting grey and tough. The tumor boundary was not clear. Fast pathology diagnosis was small round cytoma, first considering pituitary adenoma. The final pathology revealed hyperemic tissue with IHC showing MIB-1 (4%+), PLAP (-), SY (-), CD1a (-), CD138 (+), PRL (-), GH (-), ATCH (-), κ (+), λ (+), CD2 (+) sporadic, CD20 (+) sporadic and PAX5 (+) sporadic.

The patient was stable with GCS 15 and no obvious change in vision. The patient received neither radiotherapy nor chemotherapy. The patient died in a follow-up of 1.26 year.

**Case 3 Jugular foramen plasmacytoma**

Patient is a 58-year-old male and has had intermittent dizziness for approximately 2 weeks. The patient denied headache, vomiting, seizure or other cranial deficits. The examination reviewed no obvious hearing loss or cerebellar signs. CT and MRI revealed jugular foramen lesion (Supplementary figure.3).

A C-shaped incision behind left ear was designed, with the accompanying of intraoperative facial nerve monitoring, the pre-sigmoid was exposed and left jugular foramen was further drilled. The tumor located in the jugular foramen with moderate hardness and blood supply. Fast pathology showed paraganglioma. The tumor was removed piece by piece totally with lower sigmoid sinus and jugular bulb.

The final pathology presented lymphatic hematopoietic system tumor, combined with IHC enzyme marker CD138 (+), CD20 (-), CD3 (-), CgA (-), CK (-), κ (+), λ (-), MIB-1 (5%) and considering plasmacytoma. The patient showed no recurrence or metastasis, stable with no complains. The patient received chemotherapy with no evidence of multiple myeloma until now.

**Case 4 Right frontal plasmacytoma**

This 70-year-old right-handed admitted in 2015 with a 3-month history of right frontal eminence visible to naked eye. The eminence was hard and progressive enlarging. The patient complained of chest tightness and pain, but denied headache, dizziness, seizure, dyskinesia, sensory disorder or blurred vision. Results of neurological examination was negative. CT scan showed right frontal lesion with invasion of the skull. MRI demonstrated a right frontal lesion with low T1, high T2, and obvious enhancement. The pre-operation diagnosis was right frontal meningioma.

A right U-shaped incision was designed and the bone flap reached the midline. The tumor involved the entire layer of the dura mater, with invasion through the skull and into the galea aponeurotica. The tumor was soft with abundant blood supply and exogenic with no invasion of the brain. The base of the tumor was on the dura. Tumor was resected from basement piece by piece and finally subtotal removed. The frozen pathology showed small round cytoma. Considering the malignancy, the skull bone was removed.

The patient was stable and no post-operative complication occurred. Pathology showed 4*3*1cm hyperemic tissue with dura, and a 4*3*2cm skull bone with a 2*1*1cm hyperemic tumor on it. The pathology diagnosis presented right frontal plasmacytoma. The patient was diagnosis as multiple myeloma further and received no radiotherapy or chemotherapy. The reexamination a month post-operative revealed plasmacytoma recurrence. The patient died in a follow-up of 1.91 year.

**Case 5 Sphenoid ridge plasmacytoma with right temporal and orbital recurrence**

This female patient was admitted to neurosurgery department the first time in April 2010 when she was 44 with a 2-month history of right proptosis, eyelid swelling and pain. 10 days before admission she presented with aggravating headache. The patient denied seizure, dysphasia, dyskinesia or sensory disorder. Examination revealed right decreased vision of VOS 1.0 and VOD 0.8 with right temporal hemianopsia and diplopia. Right eye proptosis is obvious with poor right eyelid closure. Eye movement test revealed limited abduction of right eye. CT demonstrated a right sphenoid ridge lesion with equal signal. MRI revealed a 3*3cm right lateral sphenoid ridge lesion with low T1, high T2 and obvious enhancement. Pre-operation diagnosis based on clinical characteristics and radiology show high possibility of meningioma.

A right zygomatic pterional approach was conducted to reach the tumor. A cranioplasty was further conducted. The tumor was subtotally removed. Pathology revealed right sphenoid ridge solitary plasmacytoma. IHC showed CD138 (+), EMA (+)，κ (-), λ (-/+), CK (-), CD79a (+/-), L26 (-), CD3 (-), CD4 (-), CD8 (-), KP1 (-), S100 (-), LCA (-/+), CD1a (-), LgG (+), Cong-red (-), MIB-1 10%. After surgery, the patient had no apparently changes in vision and mRS.

The patient was admitted in hematological department. Whole body bone SPECT Imaging showed abnormal increasing of radioactivity on skull, left scapula, both articulationes sacroiliaca and left ischium. Bone marrow aspiration demonstrated 4-5 cavum medullare with abundant cell counts, accounting for 50%, visible of megakaryocyte, mature neutrophil and eosinophile granulocyte. A small amount of sporadic plasmocyte can be observed with enzyme marker showing polyclone with no obvious abnormity, demonstrating LCA partly (+), L26 (-), CD79 (-), UCHL-1 (+) sporadic, CD3 (+) individual, EMA (+) individual, CD138 (+) sporadic, CD34 (-), κ (-), λ (-), MPO (+) partly, CD56 (-), CD99 (+), CDa (-), TdT (-), Kp-1 (+) sporadic. Further examination including blood immunoelectrophoresis, serum immunoprotein and PET/CT confirmed the diagnosis of multiple myeloma. She received radiotherapy and chemotherapy of Velcade 1.75mg on d1, 4, 8 and 11, with dexamethasone 10mg on d1-4 and d8-11.

Two years postoperatively, the patient was admitted again in Feb 2012 with right frontotemporal eminence visible to naked eye. No obvious progress symptoms occurred after last surgery. CT revealed a newly presented right temporal lesion with orbital invasion. The patient received right temporal tumorectomy with the lesion total removed. The lesion was hard with hyperemic section. Later, Pathology revealed recurrent right temporal and orbital plasmacytoma. IHC demonstrated CD138 (+), EMA (+)，κ (-), λ (+), S100 (+), LCA (-), CD79a (-), MIB-1 15%. In a follow-up of 5.7 years, the patient was confirmed died with no obvious information.
