# Supplementary material for: Enhanced clinical outcomes with radiotherapy in diagnostically challenging intracranial plasmacytomas: Analysis of 190 cases
Source: Cancer Med. 2024 Mar 8;13(4):e7017. doi: 10.1002/cam4.7017 (PMC10922021; doi:10.1002/cam4.7017)
Supplement: Supplementary file 2 — Figure S1. [file CAM4-13-e7017-s002.docx]

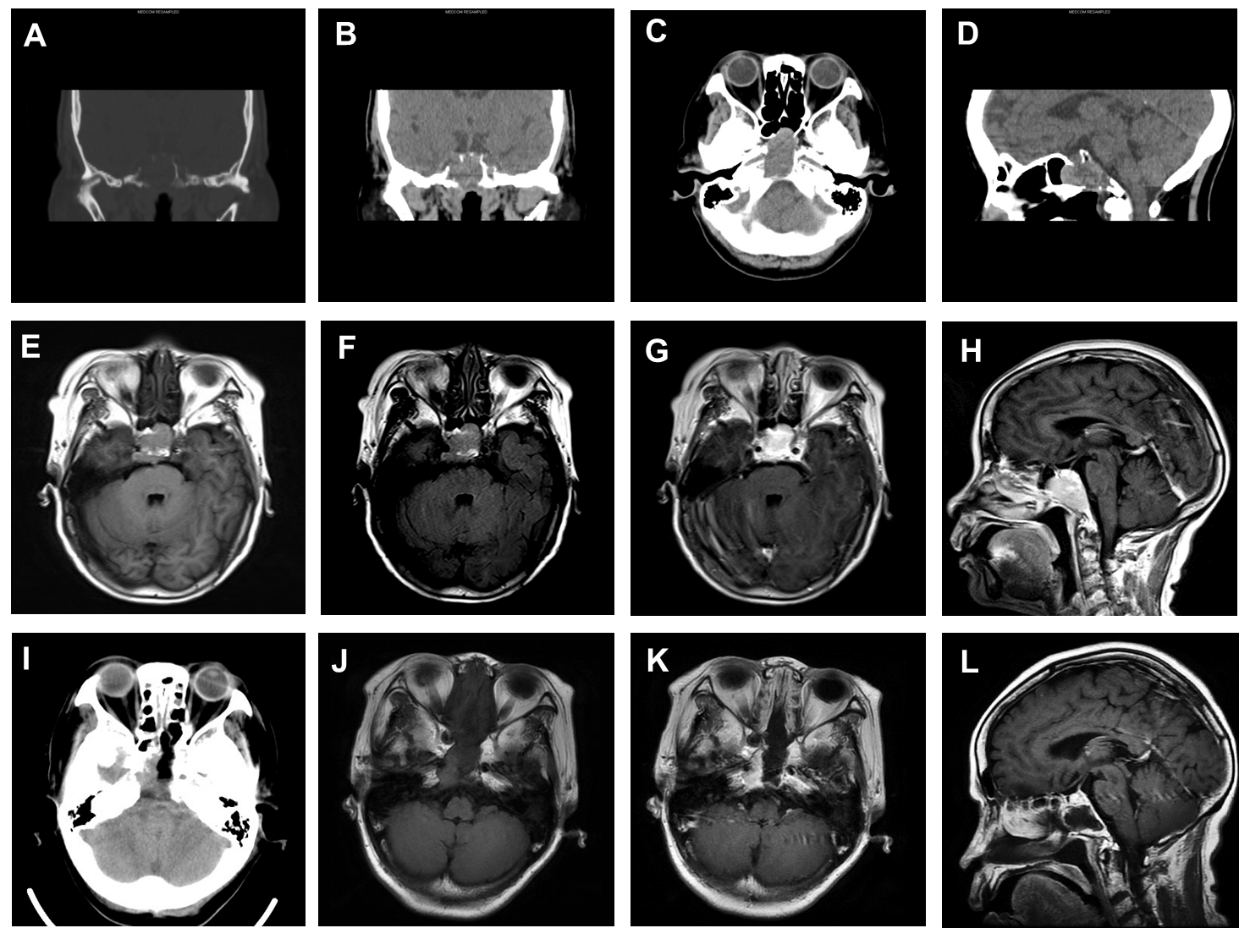


**Supplementary fig. 1** Pre-and post-operative MRI and CT of case 1

(**A**-**H**): Preoperative MRI and CT showed a clival lesion that had an isointense signal on T1 images and homogeneous enhancement after contrast with an osteolytic appearance. (**I**-**L**): Postoperative MRI and CT showed subtotal resection of the tumor.


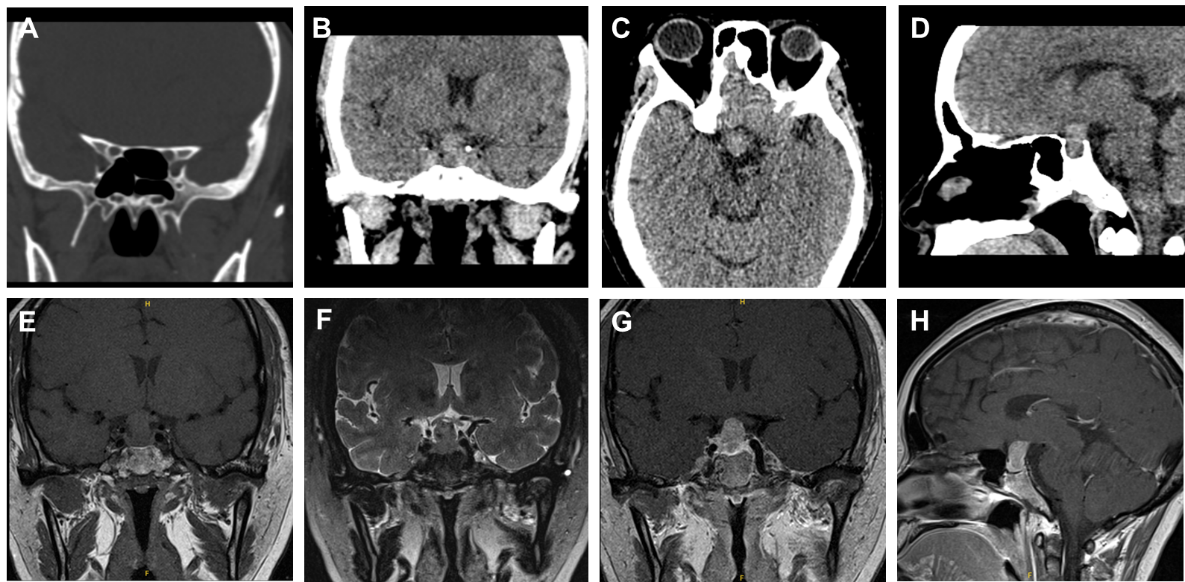


**Supplementary fig. 2** Preoperative MRI and CT of case 2

(**A**-**D**): Preoperative CT showed a sellar lesion. (**E**-**H**): Preoperative MRI revealed a sellar lesion with equal T1, equal T2 and obvious enhancement.


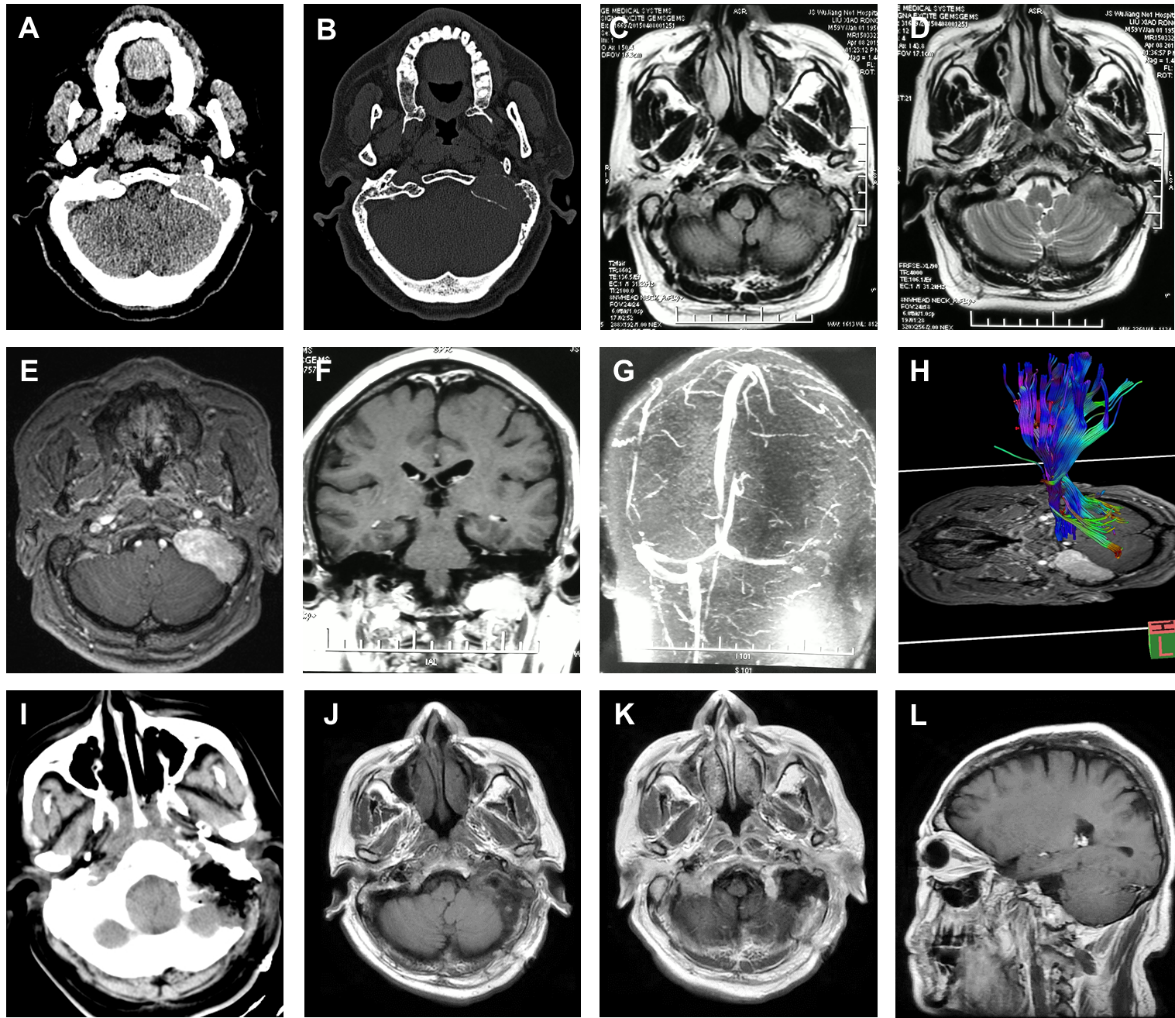


**Supplementary fig. 3** Preoperative MRI and CT of case 3

(**A**-**B**): Preoperative CT showed a jugular foramen lesion. (**C**-**F**): Preoperative MRI revealed a jugular foramen lesion with equal T1, equal T2 and obvious enhancement. (**G**): Preoperative Magnetic Resonance Venogram. (**H**): Preoperative diffusion tensor imaging. (**I-L**): Postoperative MRI and CT showed subtotal resection of the tumor.
