# Supplementary material for: Enhanced clinical outcomes with radiotherapy in diagnostically challenging intracranial plasmacytomas: Analysis of 190 cases
Source: Cancer Med. 2024 Mar 8;13(4):e7017. doi: 10.1002/cam4.7017 (PMC10922021; doi:10.1002/cam4.7017)
Supplement: Supplementary file 3 — Table S1. [file CAM4-13-e7017-s003.docx]

| **Supplement Table 1 Clinical features of intracranial plasmacytoma patients from reviewed literature** | | | | | | | | | | |
| --- | --- | --- | --- | --- | --- | --- | --- | --- | --- | --- |
| **Case** | **Age/Sex** | **Tumor location** | **Presenting signs and symptoms** | **MRI features** | **Procedure** | **Pathology & IHC stain** | **Treatment** | **MM Status** | **F/U period (months) and status** | **Reference** |
| **1** | 55, M | Clivus | Diplopia, right CNVI palsy, nystagmus on right gaze | T1 & T2 isointense, with enhancement | Resection | CD138(+), CD38(+), CD56(+), CD20(-), cytokeratin(-), synaptophysin(-), Ki67 < 5%, kappa (+), lambda (-) | RT | N | 8, alive | Amita et al, 2015 |
| **2** | 57, M | Right calvaria | H/A after accident | Enhanced calvarial mass, reduced size after 3w | Gross total resection | 8% CD138(+), CD56+, Lambda (+), Kappa (-), 30% Ki67 30% | Ch | Y, post-op | 9, alive | Morgensten et al, 2017 |
| **3** | 41, M | Left frontoparietal lobe | H/A and vomiting | Left frontoparietal lobe mass, with homogenous enhancement | Total resection | Lambda (+) | RT | N | 6, alive | Kara et al, 2016 |
| **4** | 62, F | Sellar | Right eye blurred vision, right temporal H/A | Homogenously enhancing intrasellar mass | Total resection | CD79A(+), MUM-1(+), monoclonal kappa (+) | RT | N | 18, alive | Soejbjerg et al, 2016 |
| **5** | 27, F |  | H/A and vomiting |  | Total resection | CD138(+), MUM-1(+), kappa (+), CD20(-), CD56(-) | - | Unknown | Unknown | Kumar et al, 2016 |
| **6** | 31, M | Clivus | H/A and diplopia, history of POEMS syndrome | Mass lesion of clivus, with heterogenous enhancement | Biopsy |  | Ch, RT | Unknown | Unknown | Thomas et al, 2016 |
| **7** | 69, M | Clivus | Diplopia, bilateral CNVI palsy | T1 isointense clival mass, with moderate enhancement | Resection | CD138(+), CD56(+), lambda (+), kappa (-) | RT | Y, post-radiation | 18, alive | Kalwani et al, 2015 |
| **8** | 42, F | Orbital apex | Bilateral diplopia, exophthalmos, history of orbital apex mass, left proptosis | Enhancing left-sided mass extending through left SOF | Biopsy | CD138(+), CD20(+), CD3(+), and Ki67(+) | RT | N | 16, alive | Huggins et al, 2015 |
| **9** | 47, M | Right frontal convexity | H/A, left hemiparesis, depressed mood, GTCS | Hyperintense on T1, isointense on T2, with homogenous enhancement | Total resection | Monoclonal kappa (+) | Unknown | N | 9, alive | Pour Khalili et al, 2015 |
| **10** | 56, M | Calvarial, multiple | Right tongue deviation and numbness, mild dysarthria, dysphagia | Extensive disease in the cervical and thoracic vertebra |  |  | Ch, RT | Unknown | Unknown | Sin et al, 2015 |
| **11** | 30, M | Anterior cranial fossa base | Left proptosis w/ H/A | T1 isointense, T2 slightly hyperintense, with homogenous enhancement | Total resection |  | RT | N | 15, alive | Vengalathur et al, 2014 |
| **12** | 34, M | Left frontoparietal dura | Hallucination and amnesia | Left frontotemporal extra-axial mass with broad dural base | Resection | CD38(+), MUM1(+), kappa (+), lambda (-), cytokeratin(-), vimentin(-), EMA(-), MPO(-) | RT | Unknown | Unknown | Azarpira et al, 2012 |
| **13** | 75, F | Falx cerebri | H/A, difficulty writing, forgetfulness, vertigo | Enhanced extra-axial dural-based temporal mass | Biopsy | MUM1(+), scattered CD138(+) cells, majority EMA(+), kappa (+), lambda (-) | RT | N | 60, alive | Devoe et al, 2014 |
| **14** | 45, M | Left parietal extra-axial | H/A | Extra-axial left parietal mass | Resection | CD138(+), lambda (+), | RT | Unknown | Unknown | Anoop et al, 2014 |
| **15** | 25, M | Right maxilla | H/A, right facial pain, diplopia, right RAPD, decreased right eye vision |  | Biopsy |  | Ch | N | 12, alive | Webb et al, 2011 |
| **16** | 54, F | Multiple calvarial | Multiple palpable skull masses, left hemianopia |  | Biopsy |  | Ch, RT | Unknown | Unknown | Webb et al, 2011 |
| **17** | 76, F | Calvarial, left frontoparietal | Cranial mass in left frontoparietal area, LMN signs in lower limbs |  | Biopsy |  | Ch | Unknown | Unknown | Webb et al, 2011 |
| **18** | 63, M | Right parieto-occipital extra-axial | Enlarging right parieto-occipital mass | T1 isointense, with partial enhancement | Total resection |  | Unknown | Unknown | Unknown | Gürbüz et al, 2013 |
| **19** | 58, F |  | Aggravating H/A | Extra-axial, bone-destroying mass, with hemogenous endancement | Total resection | CD18(+), kappa (+) | Ch, RT | N | 14, alive | Hasturk et al, 2012 |
| **20** | 37, M | Right p-fossa | Occipital H/A | Isointense on T1, T2 and FLAIR, with avid enhancement | Total resection | CD45(-) | RT | Unknown | Unknown | Darghighi et al, 2012 |
| **21** | 54, F | Clivus, spenoid sinus, cavernous seg. of ICA | Deteriorating right eye vision, H/A, right inferior hemianopia | Spheno-clival mass isointense on T1, T2, with homogenous enhancement | Partial resection | CD38(+), CD138(+), CD56(+), CD20(-), GFAP(-), chromogranin A (-), synaptophysin (-), Ki67 <1% | RT | N | 22, alive | Liu et al, 2010 |
| **22** | 40, M | Clivus | H/A, progressive right eye blurred vision and diplopia, right eye ptosis, impair adduction, elevation and depression, dilated sluggish right pupil | A well-circumscribed lobulated soft tissue mass arising from the clivus, bulging anteriorly into the sphenoid sinus and posteriorly into the prepontine cistern | Biopsy |  | Ch, RT | Y | 6, alive | Kashyap et al, 2010 |
| **23** | 42, F | Right p-fossa | Occipital H/A, nausea and vomiting | Highly vascular mass, centered on the squamous portion of the right occipital bone | Partial resection |  | Ch, RT | Unknown | Unknown | Patel et al, 2010 |
| **24** | 71, F | Left parietal calvarial | History of 2 TIAs | Blood in the subarachnoid space along with dural enhancement | Biopsy |  | RT | Y | 3, alive | Miles et al, 2009 |
| **25** | 59, M | Sellar, sphenoid sinus | Left eye temporal hemianopia, decreased right eye vision | Hypointense on T1, with avid enhancement | Subtotal resection | kappa (+), lambda (-), CD79a(+), Ki67 20% | RT | Unknown | Unknown | Cao et al, 2009 |
| **26** | 43, F | Left orbital apex | Progressive left eye vision deterioration, acute unilateral visual loss | Slightly hyperintense cone-shaped mass in the left optic canal, with homogenous enhancement | Resection | Lambda (+) monoclonality | Ch, RT | Y, post-op | 12, alive | Wachter et al, 2010 |
| **27** | 59, M | Right temporal region | Amnesia and gait disturbance | Right temporal mass | Partial resection | kappa (+), lambda (-), CD56(-), cyclin D1 (-) | RT | N | 12, alive | Manabe et al, 2010 |
| **28** | 81, F | Clivus | Gait disturbance | Clival mass | Resection | Kappa (+), lambda (-), CD45(-), CD20(-), cytokeratin (-), EMA(-), vimentin (-), synaptophysin (-), chromogranin (-), S100 (-), GFAP(-), Ki67 11% | Ch, RT | Y, at 6 month follow-up | 18, dead | Terada, 2009 |
| **29** | 78, F | Left parietal cranium | Right hemiparesis, left parietal mass | Isointense on T1, hyperintense on T2, with intense homogenous enhancement | Total resection |  | Ch | N | 12, alive | Zigouris et al, 2009 |
| **30** | 32, M | Sphenoid sinus, sphenoid wing | Worsening right eye pain, diplopia, right CNVI palsy | Sphenoid sinus mass, with ring enhancement | Partial resection | CD20(-), CD(68+), EMA(+), CD38(+), lambda (+), Ki67 3% | RT | N | 8, alive | Park et al, 2009 |
| **31** | 31, M | Right parietal calvarial | Symmetric motor and sensory dysfunction in lower limbs, CIDP |  | Biopsy |  | Unknown | Unknown | Unknown | Koo et al, 2009 |
| **32** | 61, F | Right frontal lobe | Gait disturbances, partial left hemiparesis bilateral blurry vision, H/A | Extra-axial mass anterior and superior to right frontal lobe with dural thickening | Partial resection | CD138(+), CD79a(+), kappa (+) | Unknown | Unknown | Unknown | Shuaipaj et al, 2008 |
| **33** | 48, M | Left sphenoid sinus | Vertigo, diplopia, left CNVI palsy, left retro-orbital pain, hypesthesia in left CNV_2_ area | Left sphenoid sinus mass invading clivus and petrous apex | Resection |  | Ch, HSCT | Unknown | 15, dead | Ko et al, 2009 |
| **34** | 67, M | Intraventricular | H/A, right limb and facial hemiparesis, change in personality | Large intraventricular mass, with homogenous enhancement | Total resection | EMA(+), clone VS38(+), CD45(-), CD3(-), CD20(-), lambda (+), kappa (-), Ki67 30% | Ch, RT | Unknown | 18, alive | Eum et al, 2009 |
| **35** | 58, M | Sellar | Diplopia, bilateral CNIV and CNVI palsy | Isointense sellar soft tissue mass eroding the clivus, with strong enhancement | Biopsy | Populations of prolactin (+) cells populations of kappa (+) w/ no overlapping | Ch, RT | Y, pre-op | Unknown | Rivera et al, 2010 |
| **36** | 70, F | Sellar | H/A, diplopia, history of pituitary adenoma surgery | Mass destroying floor of sella and invading sphenoid sinus, with strong enhancement | Resection | CD138(+), kappa (+) | Ch, RT | Y | 22, alive | Yaman et al, 2008 |
| **37** | 65, M | Sellar | H/A, right facial hypesthesia, diagnosis of nonfunctional pituitary macroadenoma | Intrasellar mass with homogenous enhancement | Biopsy | CD138(+), kappa (+) | RT | N | 4, alive | Pitini et al, 2008 |
| **38** | 65, F | Anterior skull base | Diplopia, right CNVI palsy, hypesthesia in right CNV_2_ | Extensive lobulated mass engulfing much of the skull base, with heterogenous enhancement | Total radical resection | CD38(+), lambda (+), kappa (-) | Ch, RT, HSCT | Y | 17, alive | Yamaguchi et al, 2007 |
| **39** | 64, M | Parafalcine | Right partial motor seizures, mild right hemiparesis | 2 lesions: left frontal mass adjacent to falx, and skull lesion nearby | Resection | CD45(+), CD138(+), CD20(-), CD79a(-), Pax-5(-), bcl-6(-), lambda (+), Ki67 50%, | Ch, RT | Y | 5, dead | Wavre et al, 2007 |
| **40** | 60, F | Left supraorbital | Left frontal H/A, left proptosis, epiphora with chemosis |  | Biopsy | Lambda (+), kappa (-) | Ch | Unknown | Unknown | Wein et al, 2002 |
| **41** | 48, M | Clivus | Right periorbital H/A, perioral paresthesia, recurrent epistaxis, history of esophageal atypical plasma cell tumor | Mass destroying clivus and involving multiple skull base foramina and both cavernous sinuses | Biopsy | Kappa (+), lambda (-) | Ch, RT | Y, at 1 month follow-up | 1, alive | Wein et al, 2002 |
| **42** | 48, M | Nasopharyngeal | Nasal congestion, anosmia, recurrent epistaxis, left CNVI palsy, hypesthesia in left CNV_2_ area |  | Biopsy | Kappa (+), lambda (-) | Surgical resection | N | 6, alive | Wein et al, 2002 |
| **43** | 28, F | Anterior skull base | Nasal obstruction, intermittent epistaxis, bifrontal H/A, decreasing vision, diplopia | Mass invading cavernous sinus and infratemporal fossa | Biopsy | Kappa (+), lambda (-) | RT | Unknown | Unknown | Wein et al, 2002 |
| **44** | 72, F | Right frontal dura | H/A, progressive left hemiparesis | T1 isotense, T2 hypertense right frontal mass arising from dura, with homogenous enhancement | Total resection | Kappa (+), lambda (-) | - | Y, at 3 month follow-up | 36, dead | Haegelen et al, 2006 |
| **45** | 66, F | Clivus | Frontal H/A, decreased hearing in left ear |  | Partial resection |  | - | N | 108, alive | McLaughlin et al, 2005 |
| **46** | 39, M | Clivus | Left face and arm paresthesia, intermittent diplopia, dysphagia, hoarseness | Large mass extending from ethmoid and sphenoid sinuses into clivus and petroclival junctions, invading atlantoaxial joint | Biopsy | CD38(+), kappa (+), lambda (-) | Ch, RT | Unknown | Unknown | Ustuner et al, 2003 |
| **47** | 46, M | Clivus | Left-sided hearing disturbance, unsteady gait, left CNVI & CNVII palsy, left facial hypesthesia, hoarseness, nuchal pain, head instability | Large osteolytic tumor involving clivus, bilateral petrous apices, sphenoid sinus, atlantoaxial joint, with heterogenous enhancement | Subtotal resection | Kappa (+), lambda (-), Ki67 2% | RT | N | 26, alive | Higurashi et al, 2004 |
| **48** | 53, F | Left petroclival | Diplopia, left CNVI palsy | Left petro-clival osteolytic mass involving left ICA, with homogenous enhancement | Total resection | Kappa (+), lambda (-), CD79a(+), Ki67 15% | Ch, HSCT | Y, post-op | 30, alive | Higurashi et al, 2004 |
| **49** | 62, M | Sellar | Left frontal H/A, left CNIII & CNIV palsy, left ptosis, left sluggish pupil, | Clival lesion extending into bilateral cavernous sinus, with homogenous enhancement | Resection | Kappa (+), lambda (-) | RT | Unknown | 3, alive | Lee et al, 2017 |
| **50** | 60, M | Sellar | Frontal H/A, diplopia, dizziness | Mass infiltrating into normal pituitary gland | Partial resection | Lambda (+), kappa (-) | Ch | Y, pre-op | 2, alive | Lee et al, 2017 |
| **51** | 62, M | Clivus | Diplopia, bilateral CNVI palsy, wide-based gait | Invasive clival-based mass, with heterogenous enhancement | Subtotal resection |  | Ch | Unknown | 36, alive | Lee et al, 2017 |
| **52** | 77, M | Clivus | Diplopia, right CNVI palsy | T1 hypointense clival mass, eroding clivus and sellar floor, invading pituitary gland and into cavernous sinus | Partial resection |  | Ch, RT | Y, post-op | 12, alive | Lee et al, 2017 |
| **53** | 74, F |  | Unknown |  | Biopsy | Unkown, CD138(+), light chain λ, Blastic features (-) | Unknown | Unkown | Unkown | Wilberger et al, 2016 |
| **54** | 37, F | C; occipital | No neurologic symptoms |  | Biopsy | lgG κ, CD138(+), light chain κ, Blastic features (-) | Ch | Y, post-op | 60, dead | Wilberger et al, 2016 |
| **55** | 68, M | C; cranial base | Neurologic symptoms |  | Resection | lgG κ, CD138(+), light chain κ, Blastic features (-) | Ch, RT | Y, post-op | Unknown | Wilberger et al, 2016 |
| **56** | 69, M | C; frontal | No neurologic symptoms |  | Resection | Not present, CD138(+), light chain λ, Blastic features (-) | RT | N | 120, dead | Wilberger et al, 2016 |
| **57** | 72, M | C; cranial base | Neurologic symptoms |  | Resection | lgG λ, CD138(+), light chain λ, Blastic features (-) | Ch | Y, post-op | 48, dead | Wilberger et al, 2016 |
| **58** | 61, M | C; frontal | Neurologic symptoms |  | Biopsy | lgG κ, CD138(+), light chain κ, Blastic features (-) | Ch | Y, post-op | 12, dead | Wilberger et al, 2016 |
| **59** | 35, M | C; frontal | Neurologic symptoms |  | Resection | lgG κ, CD138(+), light chain κ, Blastic features (-) | Ch, RT, HSCT | Y, post-op | 60, alive | Wilberger et al, 2016 |
| **60** | 71, F | Sellar | Neurologic symptoms |  | Biopsy | κ light chains, CD138(+), light chain κ, Blastic features (-) | Ch | Y, post-op | 36, dead | Wilberger et al, 2016 |
| **61** | 30, F | D; medulla | No neurologic symptoms |  | Biopsy | Not present, CD138(+), light chain κ, Blastic features (-) | RT | N | 48, alive | Wilberger et al, 2016 |
| **62** | 69, M | C; orbitofrontal | Neurologic symptoms |  | Resection | Unkown, CD138(+), light chain κ, Blastic features (-) | Ch, RT | Y, post-op | 48, alive | Wilberger et al, 2016 |
| **63** | 65, M | C; parieto-occipital | Neurologic symptoms |  | Biopsy | lgG κ, CD138(+), light chain κ, Blastic features (-) | Ch, RT | Y, post-op | 3, alive | Wilberger et al, 2016 |
| **64** | 57, F | Sellar, spenoid sinus | Intermittent H/A, right CNV_1_ hypesthesia, history of nonfunctional pituitary macroadenoma | Intrasellar mass with homogenous enhancement | Partial resection | CD138(+), CD43(+), kappa (+), chromogranin (-), synaptophysin (-), PLAP(-), EMA(-), prolactin (-), GH(-), ACTH(-), FSH(-), LH(-), TSH(-), | Ch, RT, HSCT | Y, post-op | 10, alive | Sinnott et al, 2006 |
| **65** | 34, F |  | H/A, diplopia, left CNVI palsy | T1 hypointense, T2 isointense irregular intracellular mass with suprasellar and parasellar extension, and heterogenous enhancement | / | CD138(+), CD20(-), kappa (+), lambda (-) | Ch, RT | Y, at admission | 48, alive | Jiang et al, 2014 |
| **66** | 51, M | Sellar | H/A, nausea, diplopia, right ptosis, weight loss | Enhancing sellar and suprasellar region invading sphenoid sinus and clivus | Partial resection | / | Ch, RT | Unknown | Unknown | Joukhadar et al, 2012 |
| **67** | 46, F | Clivus | oculomotor paralysis (diplopia), ptosis of eyelid, deviation of face, deviation of tongue | Large enhancing skull-based tumour involving the clivus and nasopharynx with extensions into the paranasal sinuses, involvement of cavernous sinuses, encasement of internal carotid arteries, extension into the orbit intracranially | Biopsy | CD138(+) | Radiation + Chemo | N | 54, alive | Khosa et al, 2017 |
| **68** | 53, F | Clivus | oculomotor paralysis (diplopia), vomiting | Lesionon the right clivus adjascent to Dorello’s canal | / | / | Radiation + Chemo | Y | 3, alive | Ibekwe et al, 2018 |
| **69** | 59, F | Posterior occipital | dizziness, dysphagia, hoarseness, aural fullness | A destructive 3.4*1.4*2.8 cm lesion of the left skull base centered within the left jugular foramen. | Biopsy | kappa(+), CD138(+) | Chemo | Y | unknown | Oushy et al, 2018 |
| **70** | 48, F | Frontoparietal | \ |  | Total resection | kappa(+) | Radiation | N | 67, alive | Ozoner et al, 2018 |
| **71** | 41, M | Central skull base | headache, oculomotor paralysis (diplopia) | A large heterogenous expansile lesion measuring 51 mm*50 mm involving the central skull base, clivus with erosion of adjacent bones, and partially encasing bilateral ICA | / | CD138(+) | Radiation | N | 12, alive | Siyag et al, 2018 |
| **72** | 60, M | Right frontal scalp(skull) | \ | T1 and T2 isointense mass | Total resection | kappa(+), CD138(+), Ki67 60% | Chemo | Y | 12, alive | Yang et al, 2018 |
| **73** | 48, M | Posterior fossa | headache, dizziness, dysgeusia | Well-limited mass, relatively homogeneous, with intense enhancement of extraparenchymal localization, pushing the left cerebellar hemisphere and compressing the 4th ventricle. | Biopsy | kappa(+), Ki67 5%, MUM-1(+) | Chemo | Y | 14, alive | Auge et al, 2019 |
| **74** | 67, F | Left parietal | headache |  | / | / | Radiation + Chemo | N | 40, dead | Chen et al, 2019 |
| **75** | 71, F | Occipital | back pain, fatigue | Dural thickening diffusely | / | / | Chemo | Y | 22, dead | Fitzgerald et al, 2019 |
| **76** | 39, M | Left middle cranial fossa | headache, amnesia, gait disturbance, deviation of face | A well-defined, partially lobulated, heterogeneously enhancing isointense left parasellar mass, measuring 5.0*4.4 cm, rising from the floor of the left middle cranial fossa | / | CD138(+) | / | Y | unknown | Gregorio et al, 2019 |
| **77** | 59, M | Clivus | oculomotor paralysis (diplopia) | T1 isointense, T2 isointense mass with homogenous enhancement | Subtotal resection | / | / | N | 128, alive | Ma et al, 2019 |
| **78** | 45, F | Orbitotemporal | headache, blurred vision | – | Subtotal resection | / | / | Y | 126, alive | Ma et al, 2019 |
| **79** | 35, M | Petrous apex | facial paresthesia, tinnitus and hearing loss, ataxia | T1 isointense, T2 hypointense mass with homogenous enhancement | Partial resection | / | Radiation | Y | 122, alive | Ma et al, 2019 |
| **80** | 42, M | Clivus | oculomotor paralysis (diplopia), facial paresthesia, tinnitus and hearing loss, deviation of face | T1 isointense, T2 hyperintense mass with homogenous enhancement | Partial resection | / | Radiation + Chemo | N | 118, alive | Ma et al, 2019 |
| **81** | 56, F | Sellar | dizziness, blurred vision | T1 mild hyperintense, T2 mild hyperintense mass with homogenous enhancement | Partial resection | / | / | N | 60, dead | Ma et al, 2019 |
| **82** | 54, M | Clivus | tinnitus and hearing loss | T1 isointense, T2 hyperintense mass with homogenous enhancement | Partial resection | / | Radiation | N | 97, alive | Ma et al, 2019 |
| **83** | 58, F | MCB | blurred vision, oculomotor paralysis (diplopia), facial paresthesia | – | Subtotal resection | / | / | Y | 41, dead | Ma et al, 2019 |
| **84** | 47, F | Clivus | oculomotor paralysis (diplopia) | T1 isointense, T2 hyperintense mass with homogenous enhancement | Total resection | / | Radiation + Chemo | Y | 28, dead | Ma et al, 2019 |
| **85** | 68, M | Frotal | \ | T1 isointense, T2 isointense mass with homogenous enhancement | Total resection | / | / | N | 58, alive | Ma et al, 2019 |
| **86** | 64, M | Clivus | oculomotor paralysis (diplopia) | T1 isointense, T2 isointense mass with homogenous enhancement | Subtotal resection | / | / | N | 52, alive | Ma et al, 2019 |
| **87** | 47, F | Clivus | blurred vision | T1 isointense, T2 hyperintense mass with homogenous enhancement | Partial resection | / | / | Y | 42, alive | Ma et al, 2019 |
| **88** | 73, F | Clivus | dizziness, oculomotor paralysis (diplopia) | T1 isointense, T2 isointense mass with homogenous enhancement | Partial resection | / | Chemo | N | 36, alive | Ma et al, 2019 |
| **89** | 55, F | Parasellar | \ | T1 hyperintense, T2 hyperintense mass with homogenous enhancement | Total resection | / | Radiation | Y | 35, alive | Ma et al, 2019 |
| **90** | 50, M | Clivus | oculomotor paralysis (diplopia) | T1 isointense, T2 iso-hyperintense mass with homogenous enhancement | Partial resection | / | Radiation | N | 33, alive | Ma et al, 2019 |
| **91** | 54, F | Clivus | headache, aphasia | T1 isointense, T2 isointense mass with homogenous enhancement | Partial resection | / | Radiation + Chemo | Y | 21, dead | Ma et al, 2019 |
| **92** | 57, F | Temporal | headache, aphasia | T1 hyperintense, T2 mild hyperintense mass with homogenous enhancement | Total resection | / | / | N | 27, alive | Ma et al, 2019 |
| **93** | 69, F | PCB | oculomotor paralysis (diplopia), deviation of face | T1 isointense, T2 isointense mass with homogenous enhancement | Total resection | / | Chemo | N | 20, alive | Ma et al, 2019 |
| **94** | 65, M | Sellar | oculomotor paralysis (diplopia) | homogenously enhanced mass involving the sphenoid sinus and clivus | biopsy | lambda(+), CD138(+) | Radiation | Y, 15 months post-op | 36, alive | Lee et al, 2021 |
| **95** | 54, M | Sellar | headache | homogeneously enhancing lesion in the sellar region, involving both the sphenoid sinus and clivus | Partial resection | kappa(+), CD138(+) | Radiation+Chemo | Y | 17, alive | Lee et al, 2021 |
| **96** | 45, M | Clivus | headache, oculomotor paralysis (diplopia), vomiting, shoulder and neck pain | extensive osseous lesions involving the clivus extending into the left sphenoid and cavernous sinus | biopsy | lambda(+) | Radiation and chemotherapy (with  carfilzomib, lenalidomide, elotuzumab and dexamethasone  combination) | Y | three cycle of chemotherapy, alive | Yazdanpanah et al, 2020 |
| **97** | 50, M | Occiput | headache | intra- and extra-cerebral expansile osseous lesion (79*47 mm); isointense on both T1- and T2-weighted images with homogenous enhancement | extended resection | kappa(+), lambda(+), CD138(+), Ki67 30% | N | unknown | 10, alive | Wang et al, 2020 |
| **98** | 50, M | Left frontal calvarial | headache, vomiting, limb weakness | homogeneously enhancing dural-based lesion in left fronto‑parietal region measuring 10.3 cm*4.9 cm | Total resection | kappa(+), CD138(+) | n | N | 12, alive | Gajaria et al, 2020 |
| **99** | /, / | Condyle, medial JF | shoulder and neck pain | heterogeneously enhanced mass | Total resection |  | / | unknown | unknown | Basma et al, 2021 |
| **100** | 36, F | Posterior cranial fossa | headache, dizziness | homogeneously enhanced mass ventral to the pons at the skull base | biopsy | lambda(+), CD138(+), MUM-1(+) | / | N | 3, alive | Khilji et al, 2022 |
| **101** | 61, F | Left frontal-parietal mass | headache, limbs paresthesia | left frontal parietal mass with compression of the adjacent brain parenchyma, T2 hyperintense with obviously enhancement | Total resection | lambda(+), CD138(+), Ki67 20+%, MUM-1(+) | bortezomib, dexamethasone, cy clophosphamide, and thalidomide | Y | eighth course of chemotherapy, alive | Guo et al, 2020 |
| **102** | 67, M | Left sphenoid sinus | oculomotor paralysis (diplopia) | T1 hypointense | biopsy | kappa(+) | N | N | 3, alive | Bonduelle et al, 2021 |
| **103** | 59, M | Parietal bone, occipital squama | headache, gait disturbance, urinary incontinence | isointense in T1-  and T2-weighted images with homogenous enhancement | Total resection | kappa(+), CD138(+), Ki67 10% | 28 fractionated craniospinal radiotherapy  sessions | N | 6, alive | Andaluz et al, 2022 |
| **104** | 50, F | Posterior fossa | headache, facial pain, gait disturbance, deviation of face | isointense in T1-weighted images and hypointense in T2-weighted images, with homogenous enhancement | Subtotal resection | lambda(+), CD138(+), Ki67 5% | fractioned radiotherapy, | N | 6, alive | Andaluz et al, 2022 |
| **105** | 61, M | right skull base centered in the occipital bone | shoulder and neck pain | T1 hypointense; T2 hyperintense; enhancing destructive mass | n | / | / | unknown | unknown | Wang et al, 2021 |
| **106** | 80, F | Posterior fossa | tinnitus and hearing loss, aural fullness | hyperintense on T2 FLAIR; isointense on T2; slight hyperintensity on T1; enhancement | / | kappa(+), CD138(+) | / | unknown | unknown | Conway et al, 2021 |
| **107** | 43, F | petrous | NA | NA | Subtotal resection | Lambda(+), Ki67 5% | RT | Y, 10 months post-op | 31, alive | Theodore et al, 2001 |
| **108** | 55, F | clivus | oculomotor paralysis, blurred vision, proptosis | NA | biopsy | Lambda(+), Ki67 5% | RT | Y | 28, alive | Theodore et al, 2001 |
| **109** | 73, F | petrous | NA | NA | biopsy | kappa(+), Ki67 5% | RT | Y | 22, alive | Theodore et al, 2001 |
| **110** | 54, F | orbital rim | NA | NA | Total resection | Lambda(+), Ki67 60% | no | Y | 4 dead | Theodore et al, 2001 |
| **111** | 82, F | frontal | NA | NA | Total resection | Lambda(+), Ki67 5% | no | Y | 24, alive | Theodore et al, 2001 |
| **112** | 77, F | falx | NA | NA | Total resection | kappa(+), Ki67 8% | RT | N | 52, alive | Theodore et al, 2001 |
| **113** | 68, F | frontal | NA | NA | biopsy | kappa(+), Ki67 6% | RT | N | 4, dead | Theodore et al, 2001 |
| **114** | 37, F | temporal | NA | NA | Total resection | Lambda(+), Ki67 5% | RT | N | 96, alive | Theodore et al, 2001 |
| **115** | 67, F | Right orbit | Proptosis, headache | homogenously enhanced | Partial resection | Lambda(+) | RT | N | 1.25, alive | Ashraf et al, 2003 |
| **116** | 49, M | frontal | headache | T1 hyperintense, T2 hyperintense, avidly and homogenously enhanced | Subtotal resection | NA | RT and CMT | N | 24, alive | Ertugrul et al, 2003 |
| **117** | 61, F | orbit | Headache, blurred vision, facial numb | avidly and homogenously enhanced | Partial resection | kappa(+) | RT | N | 12, alive | Brannan et al, 2003 |
| **118** | 50, M | clivus | Headache, blurred vision | T1 isointense, T2 isointense | biopsy | kappa(+) | RT and CMT | N | 8, alive | Brannan et al, 2003 |
| **119** | 24, M | Left frontal | painless mass | T1 isointense, T2 hypointense, homogenously enhanced | Total resection | NA | RT | N | 24, alive | Lee et al, 2004 |
| **120** | 59, F | frontal | headache | avidly and homogenously enhanced | Total resection | Lambda(+) | RT | N | NA | GALLINA et al, 2004 |
| **121** | 62, M | frontal | Headache, dizziness, vomit | NA | Subtotal resection | NA | RT | N | 24, alive | MITSOS et al, 2003 |
| **122** | 67, F | frontal | Limb weakness | T1 hyperintense, T2 hypointense, avidly and homogenously enhanced | Total resection | λ(-) κ(+) | NA | N | 45, alive | Cerase et al, 2008 |
| **123** | 79, M | frontal | headache | T1 isointense, T2 isointense, avidly and homogenously enhanced | NA | λ(-) κ(+) | NA | N | 12, alive | Cerase et al, 2008 |
| **124** | 72, M | Temporal, orbit | Dizziness, limb weakness | NA | NA | λ(-) κ(+) | NA | Y | NA | Cerase et al, 2008 |
| **125** | 82, M | cavernous sinus region | oculomotor paralysis, blurred vision | T1 isointense, T2 isointense, moderate and homogenously enhanced | biopsy | λ(-) κ(+) | CMT | Y | 6, alive | Cerase et al, 2008 |
| **126** | 60, M | frontal | Headache, gait disturbance | heterogenously enhanced | NA | NA | RT and CMT | Y | 114, dead | Chambless et al, 2010 |
| **127** | 77, M | Frontal, occipital, parietal | Aphasia, limb weakness | T1 hyperintense, T2 hyperintense, avidly and homogenously enhanced | biopsy | NA | RT | N | 1, dead | Duerinck et al, 2012 |
| **128** | 40, M | Temporal, sphenoid ridge, orbit | oculomotor paralysis, blurred vision | NA | biopsy | NA | NA | N | NA | GANGADHAR et al, 2012 |
| **129** | 50, F | Sella, clivus | headache, blurred vision | T1 isointense, T2 hyperintense, avidly and heterogenously enhanced | Total resection | λ(-) κ(+) | RT | N | 165, alive | Gagliardi et al, 2014 |
| **130** | 68, M | clivus | headache, blurred vision | T1 isointense, T2 hyperintense, avidly and homogenously enhanced | Partial resection | λ(-) κ(+) | RT and CMT | Y, 13 months post-op | 22, dead | Gagliardi et al, 2014 |
| **131** | 57, M | clivus | headache | T1 isointense, T2 hyperintense, avidly and heterogenously enhanced | Subtotal resection | λ(-) κ(+) | RT | N | 20, alive | Gagliardi et al, 2014 |
| **132** | 53, F | Sella, clivus | blurred vision | T1 isointense, T2 hyperintense, avidly and homogenously enhanced | Total resection | λ(-) κ(+) | RT | N | 9, alive | Gagliardi et al, 2014 |
| **133** | 46, M | clivus | facial numbness | T1 hyperintense, T2 hypointense, homogenously enhanced | Subtotal resection | λ(+) κ(-) | RT | N | 48, alive | KDODWEDUL et al, 2014 |
| **134** | 84, F | clivus | blurred vision, headache | T1 hyperintense, avidly and heterogenously enhanced | biopsy | λ(+) κ(-) | RT and CMT | Y | NA | Rahman et al, 2016 |
| **135** | 36, M | frontal | headache | T1 hyporintense, T2 hyperintense, heterogenously enhanced | NA | NA | RT | N | 3, alive | Mankotia et al, 2017 |
| **136** | 37, M | frontal | Limb weakness | NA | NA | NA | RT | N | 26, alive | Wiśniewski et al, 2018 |
| **137** | 50, M | clivus | blurred vision, headache | T1 hypointense, T2 isointense, avidly and homogenously enhanced | Subtotal resection | λ(-) κ(+) | RT | N | 29, alive | Jin et al, 2018 |
| **138** | 59, F | Sella, clivus | oculomotor paralysis, blurred vision, headache | T1 isointense, moderate and homogenously enhanced | Subtotal resection | NA | RT | N | 15, alive | Jin et al, 2018 |
| **139** | 61, M | clivus | blurred vision, headache | T1 isointense, homogenously enhanced | Subtotal resection | λ(-) κ(+) | RT and CMT | N | 120, alive | Jin et al, 2018 |
| **140** | 53, M | clivus | blurred vision, headache | avidly and heterogenously enhanced | Subtotal resection | λ(-) κ(+) | RT | N | 14, alive | Jin et al, 2018 |
| **141** | 47, F | Sella, clivus | oculomotor paralysis, blurred vision, headache, limb weakness, gait disturbance | heterogenously enhanced | Subtotal resection | λ(+) κ(-) | RT and CMT | N | 24, alive | Jin et al, 2018 |
| **142** | 30, NA | sphenoid ridge, orbit | blurred vision, headache | T2 hypointense, homogenously enhanced | Partial resection | λ(+) κ(-) | RT | Y, 48 months post-op | 84, alive | Dang et al, 2019 |
| **143** | 43, M | Sella, clivus | headache | T1 hypointense, T2 hypointense | biopsy | NA | neither | Y | 4, dead | Shankar et al, 2020 |
| **144** | 33, F | orbit | Dizziness, facial numbness | T1 hypointense, moderate homogenously enhanced | Subtotal resection | λ(+) κ(+) | RT and CMT | N | 16, alive | Yanagihara et al, 2020 |
| **145** | 60, M | sella | blurred vision | T1 hypointense, T2 hypointense, moderate homogenously enhanced | biopsy | λ(+) κ(-) | RT and CMT | Y | 1, alive | Waqar et al, 2022 |
| **146** | 54, F | Frontal, parietal | blurred vision, headache | T1 hypointense, T2 hyperintense | NA | λ(+) κ(-) | RT | N | 36, alive | Sato et al, 2022 |
| **147** | 54, F | temporal | blurred vision, headache | avidly homogenously enhanced | biopsy | λ(-) κ(+) | RT and CMT | N | 16, alive | Meinhardt et al, 2022 |
| **148** | 52, M | sella | blurred vision, headache | T2 hypointense | NA | λ(+) κ(-) | RT and CMT | Y | alive | Johnson et al, 2022 |
| **149** | 75, F | right parietal region | no | avidly homogenously enhanced | Total resection | λ(-) κ(+) | neither | N | alive | Naganuma et al, 2004 |
| **150** | 58, F | occipital region | no | avidly homogenously enhanced | Total resection | λ(-) κ(+) | RT and CMT | Y, 3 months post-op | alive | Naganuma et al, 2004 |
| **151** | 50, F | Left temporal-insular lobe and thalamus | motor aphasia | T2 hypointense | biopsy | λ(-) κ(+) | RT | N | alive | Ferrari et al, 2012 |
| **152** | 65, M | Clivus, sella | headaches and  progressive diplopia. | avidly homogenously enhanced | biopsy | λ(-) κ(+) | RT and CMT | Y, 9 months post-op | alive | Alafaci et al, 2012 |
